# Are There Any Significant Differences in Terms of Age and Sex in Pedestrian and Cyclist Accidents?

**DOI:** 10.3389/fbioe.2021.677952

**Published:** 2021-05-24

**Authors:** Christoph Leo, Maria C. Rizzi, Niels M. Bos, Ragnhild J. Davidse, Astrid Linder, Ernst Tomasch, Corina Klug

**Affiliations:** ^1^Vehicle Safety Institute, Graz University of Technology, Graz, Austria; ^2^Swedish National Road and Transport Research Institute, VTI, Gothenburg, Sweden; ^3^SWOV Institute for Road Safety Research, The Hague, Netherlands; ^4^Mechanics and Maritime Science, Chalmers University, Gothenburg, Sweden

**Keywords:** pedestrian, cyclists, epidemiology, injuries, sex-specific differences

## Abstract

This study has analyzed sex-specific differences in pedestrian and cyclist accidents involving passenger cars. The most frequently injured body regions, types of injuries, which show sex-specific differences and the general accident parameters of females and males were compared. Accident data from three different European countries (Austria, Netherlands, Sweden) were analyzed. The current analysis shows that for both, females and males, pedestrian and cyclist injuries are sustained mainly to the body regions head, thorax, upper extremities and lower extremities. The results show that the odds for sustaining skeletal injuries to the lower extremities (incl. pelvis) in females are significantly higher. It was observed in all datasets, that the odds of females being involved in a rural accident or an accident at night are lower than for males. Elderly pedestrian and cyclist (≥60YO) tend to sustain more severe injuries (AIS2+ and AIS3+) than younger pedestrian and cyclists (<60YO) in some of the datasets. The findings of this study highlight the differences in males and females in both, accident scenarios and sustained injuries. Further investigations are needed to distinguish between gender- and sex-specific differences causing the different injury patterns.

## Introduction

Worldwide, more than 50% of the 1.35 M road users killed annually, are vulnerable road users (VRUs) such as pedestrians, cyclists and motorcyclists (World Health Organization, 2018). Together, pedestrians and cyclists accounted for 32% of the road fatalities in the European Union in 2016 (World Health Organization, 2018). To reduce this number, a detailed analysis of the injuries is required to understand which injuries are the most common, related injury mechanisms, and finally to determine protective measures.

Awareness of sex and age specific differences in injury risks for vehicle occupants has only been identified in recent years (Kullgren and Krafft, 2010; Forman et al., 2019; Mitchell and Cameron, 2020). This may be due to the fact that vehicle safety regulations for occupants and VRUs are predominantly focused on mid-sized adult males (Simms and Wood, 2009; Linder and Svedberg, 2019; Linder and Svensson, 2019). Studies have shown that this leads to unequal treatment in terms of vehicle safety based on sex and, as a result to significant differences in the injuries sustained by males and females (Bose et al., 2011; Starnes et al., 2011; Forman et al., 2019; Leo et al., 2019b; Linder and Svedberg, 2019; Mitchell and Cameron, 2020). Starnes et al. found for example in their study that younger males (15–55 years) were significantly more likely to suffer tibia fractures than females.

Besides sex, age has also been identified as an important factor affecting the types and severity of injuries (Davis, 2001; Niebuhr et al., 2016; Leo et al., 2019a; Saadé et al., 2020). Davis and Niebuhr et al. conclude that elderly pedestrians (≥60YO) tend to suffer more severe injuries than younger pedestrians. Also Saadé et al. conclude in their study that the pedestrian age as well as the collision speed have a statistically significant influence on injuries. Especially the age group 61+ shows statistically significant differences in that study.

Anthropometric test devices (ATDs) and Human Body Models (HBMs) used for safety evaluations have been predominantly designed to match mid-sized adult males (or in rare cases small adult females). This has led to an unequal treatment of the sexes with regard to vehicle safety regulations (Linder et al., 2020). Virtual testing (VT) will play an essential role in overcoming the unequal treatment, based on sex, in vehicle safety regulations in the near future. By means of VT it is possible to assess a much larger number of test scenarios than in physical testing. Furthermore, facilitated by state-of-the-art Human Body Models (HBMs), it is also possible to implement different anthropometries and gender specific characteristics in the loop of virtual testing. As a first step, an average female anthropometry could be considered for safety evaluations, as originally proposed by Schneider (1983). Furthermore, HBMs could be even used to generate a population of HBMs representing different statures, body mass indexes and ages by applying morphing algorithms (Zhang et al., 2017).

The development of a state-of-the-art mid-sized adult female HBM and a midsized male counterpart, is one of the main objectives of the European funded VIRTUAL project (Linder et al., 2020). Knowledge of which injuries to predict, is of utmost importance for the development of such a model.

Therefore, the current study was carried out to investigate the frequency of injury types and different body regions involved for females and males. In contrast to other studies focused on vehicle occupants (Pipkorn et al., 2020), the current study focuses on pedestrians and cyclists in collisions involving passenger cars.

In two previous studies (Leo et al., 2019a, b), some initial investigations on differences in injury patterns have been performed. In the current study, these initial findings are being further discussed. An additional dataset has been included and additional parameters were analyzed to gain a better understanding of the observed differences.

This study aimed to analyze the most frequently injured body regions, which type of injuries show sex-specific differences and compare the general accident parameters, i.e., collision speed, between females and males among pedestrians and cyclists in collisions involving passenger cars.

## Materials and Methods

### Accident Data

This study is based on accident data from three different countries (Austria, Netherlands, Sweden), extracted from three different databases, for which the full abbreviated injury scale (AIS) codes of pedestrians and cyclists were available. All three databases hold data of accidents with different injury severities as well as fatalities. As the three databases differ significantly, the data of each was handled separately, and the method applied to each database as well as the results have been presented per dataset. A summary of the used data is provided in Table 1.

**TABLE 1:** Summary of accident data used for injury analyses.

	Austria		Netherland		Sweden	
			
	Accidents	Injuries	Accidents	Injuries	Accidents	Injuries
Pedestrian	308	1,083	5,272	10,436	1,311	3,182
Cyclist	144	289	15,650	29,515	1,932	3,829
Years of Recoding	2003–2019		2000–2014		2016–2018	
Filtering Criteria	− vehicle is a passenger car or van (mass up to 3.5t);		− vehicle is a passenger car or van (mass up to 3.5t).		− vehicle is a passenger car or van (mass up to 3.5t); − accidents including both a police report and a hospital report.	
	− pedestrian or cyclist was struck by only one vehicle;					
	− only one pedestrian or cyclist was involved;					
	− AIS 2005 information is available for pedestrian or cyclist;					
	− Only the first impact was taken into consideration.					
AIS version	AIS2005		AIS1990 (converted to AIS2005 using the AIS Crosswalk)		AIS2005	

#### Swedish Accident Data

The Swedish Traffic Accident Data Acquisition (STRADA) database contains information related to police reported road traffic accidents occurring on public roads in Sweden. Since its inception in 1999, the data held on STRADA has continuously increased. As of 2016, all emergency care hospitals in Sweden are included, allowing the data to be considered as nationally representative (Swedish Government Offices, 1965; Mattsson and Ungerbäck, 2013). The information provided by the police includes information about the accident location and other circumstances, i.e., date and time of accident, weather and road conditions, and posted speed limit. Hospital reports normally include a number of parameters regarding accident circumstances, i.e., a brief description of the accident, accident type and location of the accident, as well as personal information about the patient, i.e., age, gender, use of protective equipment, etc., and full diagnosis classified according to the 2005 AIS (AAAM, 2005) and the International Classification of Disease (ICD-10-SE) (AAAM, 2005; National Board of Health and Welfare, 2010). A unique aspect of the STRADA database is that police and hospital reports can be matched. Matching police and hospital reports for the same accident is of particular value in accident analysis as it allows connecting important accident circumstances (provided by the police) with details of injuries sustained in the accident (provided by the hospital). Around 30% of all accidents in STRADA include both a police and hospital report (Yamazaki, 2018). For a detailed description of the STRADA database, please see Howard and Linder (2014) and Yamazaki (2018).

The present study comprises accidents in which a cyclist or pedestrian have been injured in an accident involving a passenger car in 2016–2018. Only accidents including both a police report and a hospital report were selected. This selection resulted in 1,311 pedestrians with a total of 3,182 injuries and 1,932 cyclists with a total of 3,829 injuries.

#### Dutch Accident Data

All road traffic accidents in the Netherlands recorded by the police are included in the national road accident registration (BRON) database. BRON contains a large number of characteristics of each accident and driver as well as any involved casualties. However, police assessment of accident severity is not always accurate. Therefore, the Dutch Institute for Road Safety Research (SWOV) supplements BRON data with data from the National Basic Register Hospital Care (LBZ). This results in more reliable information of the actual severity of injuries sustained in traffic accidents. In LBZ, injuries are registered according to the ICDICD9 or ICD10, the latter since 2012. SWOV recodes these injuries into AIS90-codes using the software program ICDmap90 (SWOV, 2016). The data provided to this study contain the number of injuries in the Netherlands in 2000–2014 per AIS code according to AIS90 (using recode from ICD9/ICD10). The injuries coded according to AIS90 were converted to AIS2005-Update2008 using the AIS Crosswalk which can be used to convert injuries coded in one AIS version to another version. For 2000–2011, only injuries of patients reported in both police registration and hospital data were included, and only when road user type (pedestrian or cyclist) and opponent (car) were identical in both databases. In more recent years (2012–2014), hospitals have been using ICD10-coding which provides more extensive information on road user type and opponent. Therefore, for this particular period, injuries registered by hospitals only, have also been included. Since passenger cars and light goods vehicles are in the same category in ICD10, it cannot be guaranteed that all opponents were passenger cars.

The data from the Netherlands included cases from 2000 to 2014. These data were available for 5,272 pedestrians with a total of 10,436 injuries and for 15,650 cyclists with a total of 29,515 injuries.

#### Austrian Accident Data

The Central Database for In-Depth Accident Study (CEDATU) is an in-depth database provided by the Vehicle Safety Institute at Graz University of Technology in Austria, currently covering approximately 3,300 cases. The database includes a detailed description of accidents in Austria. Accidents with at least one injured road user are included, for which access to the court file is granted. The dataset contains accidents with fatal, serious and slight injuries. Detailed accident parameters, such as collision velocities and pre-crash trajectories are derived from accident reconstructions. Each accident case contains a set of approximately 350 core parameters. Accident parameters such as accident type, accident site, road users, etc., can be used to extrapolate findings to the national level (Tomasch and Steffan, 2006; Tomasch et al., 2008).

The following filter criteria were used to obtain the accident data set for the current study:

• vehicle is a passenger car or van (mass up to 3.5t);
• pedestrian or cyclist was struck by only one vehicle;
• only one pedestrian or cyclist was involved;
• AIS 2005 information is available for pedestrians or cyclists;
• only the first impact was taken into consideration.

These filter criteria data were available and applied for 308 pedestrians with a total of 1,083 injuries and for 144 cyclists with a total of 289 injuries. The obtained dataset includes cases from 2003 to 2019 in Austria.

### Accident Data Analysis

For analyzing differences in injuries sustained by males and females, the datasets were categorized by sex. To avoid mixing up age and sex-specific differences, injuries sustained by pedestrians or cyclists younger than 60 years old (YO) and those equal or older than 60 YO, were analyzed separately. Previous studies have shown that for pedestrian-to-passenger car collisions, elderly pedestrians (≥60YO) tend to suffer more severe injuries than younger pedestrians (<60YO) (Davis, 2001; Niebuhr et al., 2016; Saadé et al., 2020). This is another reason for splitting pedestrian as well as cyclist data for these two age groups. For all analyses, the odds-ratio (OR) and its 95% confidence interval (95%-Cl), as well as the p-value of the chi-square test, were calculated (McHugh, 2009; Szumilas, 2010; Andrade, 2015). As significance level for the *p*-value, 5% was chosen. The OR is thereby defined as the ratio of the frequency of its occurrence to the frequency of its non-occurrence (Andrade, 2015). For the current study the OR is defined as given in Equation 1, where *n*_*specific injury*_ is the number of observations for a specific injury (e.g., head injuries, femur injuries, …) for females or males and *n*_*injuries*_ is the total number of observed injuries for females or males.

Equation 1 Calculation of OR for the current study:

$$O\,R=\frac{\frac{n_{s\,p\,e\,c\,i\,f\,i\,c\,i\,n\,j\,u\,r\,y\,f\,e\,m\,a\,l\,e}}{n_{i\,n\,j\,u\,r\,i\,e\,s\,f\,e\,m\,a\,l\,e}-n_{s\,p\,e\,c\,i\,f\,i\,c\,i\,n\,j\,u\,r\,y\,f\,e\,m\,a\,l\,e}}}{\frac{n_{s\,p\,e\,c\,i\,f\,i\,c\,i\,n\,j\,u\,r\,y\,m\,a\,l\,e}}{n_{i\,n\,j\,u\,r\,i\,e\,s\,m\,a\,l\,e}-n_{s\,p\,e\,c\,i\,f\,i\,c\,i\,n\,j\,u\,r\,y\,m\,a\,l\,e}}}$$

Minor injuries (AIS1) have not been included in the current analyses, as more severe injuries are the focus of the current study. For the analysis of the most frequent AIS2+ and AIS3+ injuries, the AIS code was grouped according to the anatomical structure, e.g., skeletal, internal organ, vessels, and if possible, according to a special organ or bone, e.g., femur, tibia, lung, heart. The different anatomical structures, coded organs and bones can be found in the AIS 2005 code book (AAAM, 2005).

The results are presented in the form of tables. In order to obtain a quick and clear overview, a forest plot was integrated into the tables. An example of this visualization is shown in Table 2. The vertical gray dashed line identifies an OR of 1. The red point displays the specific OR value and the whiskers show the 95% confidence interval. In Example 1, the red point lies on the dashed gray line, which means that the observed OR value is 1 and none of the analyzed groups has higher or lower odds. For Example 2, the OR as well as the full 95%-CI has been shifted to the right of the dashed gray line. For the current study, this would mean that females have significantly higher odds of sustaining such an injury. In Example 3, on the other hand, an example showing the opposite trend can be seen, where males have significantly higher odds. In Example 4, only the OR value is shifted to the left, however, the 95%-CI overlays the gray dashed line. This means that the odds for men are higher, although not significantly so.

**TABLE 2:** Exemplary visualization of the statistical analyses.

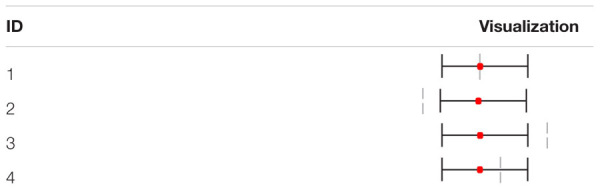

The Austrian data also provide access to other accident parameters, such as collision speeds, accident locations and road conditions, facilitating detailed investigation and gaining an insight into the type of injuries males and females are exposed to in accidents involving passenger cars. The collision speed (speed at first contact for each participant) in the database are determined using the accident reconstruction software PC-Crash (Tomasch and Steffan, 2006). Due to the nature of accident databases, this information was unfortunately not available for the Dutch and Swedish databases. The in-depth dataset of Austrian accident data was only split by sex due to the low number of accidents for some parameters following age categorization of the datasets. The results of this analysis are displayed in the form of boxplots. The number of analyzed accidents may differ for this in-depth analysis, due to certain parameters lacking for some accidents. Only accidents for which all parameters to be evaluated have been completed have been used for this analysis.

## Results

### Analysis of Injured Body Regions According AIS Classification

In Table 3, the share of injuries according the different AIS body regions for all three databases is shown. This table gives an overview of the most frequently injured body regions in all three databases for pedestrians and cyclists together.

**TABLE 3:** Share of injured body regions for AIS2+ and AIS3+ in the three different databases for pedestrians and cyclists together.

	AIS 2+						AIS 3+					
		
Body Region	Austria		Netherlands		Sweden		Austria		Netherlands		Sweden	
						
	Male	Female	Male	Female	Male	Female	Male	Female	Male	Female	Male	Female
Head	**42%**	**31%**	28%	25%	15%	11%	**63%**	**47%**	**63%**	**59%**	**31%**	**29%**
Face	3%	1%	1%	1%	6%	5%	0%	1%	0%	0%	1%	2%
Neck	1%	0%	0%	0%	1%	0%	3%	0%	3%	0%	1%	0%
Thorax	11%	14%	13%	9%	15%	10%	15%	21%	15%	8%	31%	26%
Abdomen	5%	6%	2%	2%	2%	3%	6%	7%	6%	2%	3%	5%
Spine	11%	10%	7%	7%	12%	10%	7%	4%	7%	2%	6%	6%
Upper Extremities	12%	14%	11%	10%	21%	24%	0%	3%	0%	0%	1%	2%
Lower Extremities	15%	25%	**39%**	**48%**	**30%**	**39%**	7%	19%	7%	30%	27%	30%

*Bold values indicate that most frequently injured body region of each database.*

#### Austrian Accident Data

In Supplementary Figure 1, the injured body regions as a function of sex and injury severity in Austria are displayed. The *p*-values and OR of all body regions are summarized in Table 4.

**TABLE 4:** Share of injured body regions for AIS2+ and AIS3+ injuries, OR and *p*-value in Austria for pedestrians and cyclists < 60YO and ≥ 60YO (*p-value < 5%).

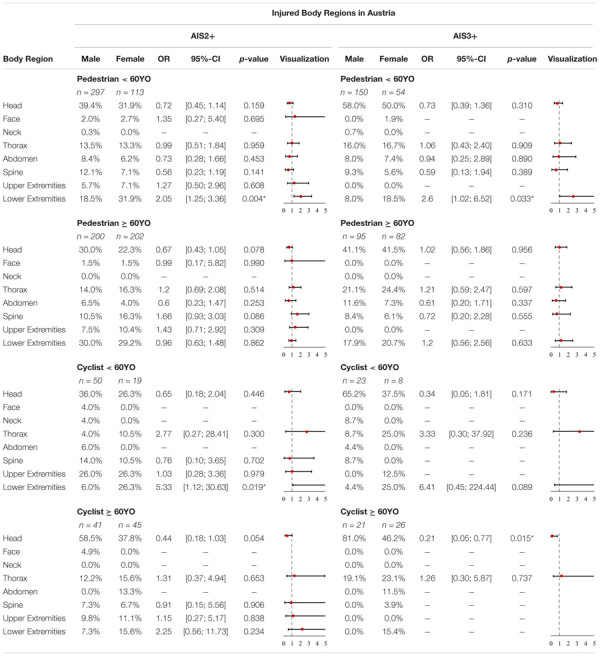

Analyzing AIS2+ injuries revealed that the three most commonly injured body regions for female pedestrians < 60YO are the lower extremities (31.9%) and head (31.9%), respectively, and the thorax (13.3%). For male pedestrians < 60YO, the three most commonly injured AIS2+ body regions are the head (39.4%) followed by the lower extremities (18.5%) and the thorax (13.5%). These statistics change when observing more severe AIS3+ injuries. Here female pedestrians < 60YO most often sustain head injuries (50%) followed by lower extremity injuries (18.5%) and injuries to the thorax (16.7%). For AIS3+ injuries, male pedestrians < 60YO most often sustain head injuries (58%) followed by thorax injuries (16%) and injuries to the spine (9.3%). Analyzing significant differences for AIS2+ and AIS3+ injuries revealed significant differences between females and males with regard to injured body regions for pedestrians < 60YO. Hence, the odds for females sustaining AIS2+ (OR = 2.05, *p*-value = 0.004) and AIS3+ (OR = 2.6, *p*-value = 0.033) lower extremity injuries are significantly higher. For other body regions, no significant differences were observed.

For female pedestrians ≥ 60YO, the three most commonly injured AIS2+ body regions are the lower extremities (29.2%) followed by the head (22.3%) and the thorax (16.3%). For male pedestrians ≥ 60YO, the three most commonly injured AIS2+ body regions are the lower extremities (30%) and head (30%), respectively, followed by the thorax (14%) and the spine (10.5%). For AIS3+ injuries we have observed the following order for females: head (41.5%) followed by thorax (24.4%) and lower extremities (20.7%). For AIS3+ injuries we have observed the following order for males: head (41.1%) followed by thorax (21.1%) and lower extremities (17.9%). Analyzing significant differences for AIS2+ and AIS 3+ did not reveal any significant differences between females and males in the Austrian data with regard to injured body regions for pedestrians ≥ 60YO.

Analyzing AIS2+ injuries, the three most commonly injured body regions for female cyclists < 60YO are the lower extremities (26.3%) followed by the upper extremities (26.3%) and the head (26.3%). For male cyclists < 60YO, the three most commonly injured AIS2+ body regions are the head (36%) followed by the upper extremities (26%) and the spine (14%). These statistics change when observing more severe AIS3+ injuries. Here female cyclists < 60YO most often sustain head injuries (37.5%) followed by lower extremity injuries (25%) and injuries to the thorax (25%). For AIS3+ injuries, male cyclists < 60YO most often sustain head injuries (65.2%) followed by thorax injuries (8.7%), injuries to the spine (8.7%) and neck injuries (8.7%). Analyzing significant differences for AIS2+ injuries of the lower extremities revealed significant differences between females and males with regard to injured body regions for cyclists < 60YO. Females have higher odds of suffering AIS2+ (OR = 5.33, *p*-value = 0.019) injuries of the lower extremities. For other body regions, no significant differences can be observed.

For female cyclists ≥ 60YO, the three most commonly injured AIS2+ body regions were the head (37.8%) followed by the lower extremities (15.6%) and thorax (15.6%), respectively, and the upper extremities (11.1%). For male cyclists ≥ 60YO, the three most commonly injured AIS2+ body regions are the head (58.5%) followed by the thorax (12.2%) and the upper extremities (9.8%). For AIS3+ injuries we observed the following order for females: head (46.2%) followed by thorax (23.1%) and lower extremities (15.4%). For AIS3+ injuries we observed the following order for males: head (81%) followed by thorax (19%). Analyzing significant differences revealed certain differences between females and males for AIS3+ with regard to injured body regions for cyclists ≥ 60YO. Males have significantly higher odds (OR = 0.21, *p*-value = 0.015) of sustaining AIS3+ head injuries. For other body regions or AIS2+ injuries, no significant differences were observed.

#### Dutch Accident Data

In Supplementary Figure 2, the injured body regions as a function of sex and injury severity in the Netherlands are displayed. The *p*-values and OR of all body regions are summarized in Table 5.

**TABLE 5:** Share of injured body regions for AIS2+ and AIS3+ injuries, OR and *p*-value in the Netherlands for pedestrians and cyclists < 60YO and ≥ 60YO (*p-value < 5%).

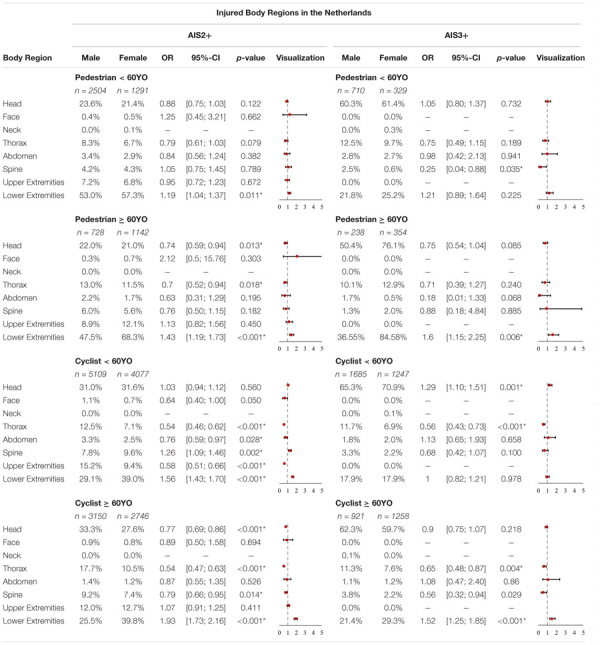

Analysis of the AIS2+ injuries revealed that the most commonly injured body regions for female pedestrians < 60YO are the lower extremities (57.3%) followed by the head (21.4%), the upper extremities (6.8%) and thorax (6.7%), respectively. For male pedestrians < 60YO the three most commonly injured AIS2+ body regions are the lower extremities (53%) followed by the head (23.6%) and the thorax (8.3%). These statistics change when considering more severe AIS3+ injuries. Here female pedestrians < 60YO most often suffer head injuries (61.4%) followed by lower extremity injuries (25.5%) and injuries to the thorax (9.7%). For AIS3+ injuries, male pedestrians < 60YO most often suffer head injuries (60.3%) followed by lower extremity injuries (21.8%) and injuries to the thorax (12.5%). Analyzing significant differences between females and males revealed significant differences for AIS2+ and AIS3+ injuries with regard to injured body regions for pedestrians < 60YO. Females have significantly higher odds (OR = 1.19, *p*-value = 0.011) of sustaining AIS2+ lower extremity injuries while the odds of males sustaining AIS3+ spinal injuries (OR = 0.25, *p*-value = 0.035) is significantly higher.

For female pedestrians ≥ 60YO, the three most commonly injured AIS2+ body regions are the lower extremities (56.5%) followed by the head (17.3%) and the upper extremities (10%). For male pedestrians ≥ 60YO, the three most commonly injured AIS2+ body regions are the lower extremities (47.5%) followed by the head (22%) and the thorax (13%). For AIS3+ injuries we have observed the following order for females: lower extremities (48%) followed by head (43.2%) and thorax (7.3%). For AIS3+ injuries we have observed the following order for males: head (50.4%) followed by lower extremities (36.6%) and thorax (10.1%). Analyzing significant differences revealed certain differences between females and males for AIS2+ and AIS 3+ with regard to injured body regions for pedestrians ≥ 60YO. Females have higher odds of suffering AIS2+ (OR = 1.43, *p*-value < 0.001) or AIS3+ (OR = 1.6, *p*-value = 0.006) injuries of the lower extremities while the odds are significantly higher for males sustaining AIS2+ head injuries (OR = 0.74, *p*-value = 0.013) and AIS2+ thorax injuries (OR = 0.7, *p*-value = 0.018).

Analyzing the AIS2+ injuries revealed that the three most commonly injured body regions for female cyclists < 60YO are the lower extremities (39%) followed by the head (31.6%) and the spine (9.6%) and upper extremities (9.4%), respectively. For male cyclists < 60YO the three most commonly injured AIS2+ body regions are the head (31%) followed by the lower extremities (29.1%) and the upper extremities (15.2%). These statistics change when considering more severe AIS3+ injuries. Here female cyclists < 60YO most often suffer head injuries (70.9%) followed by lower extremity injuries (17.9%) and injuries to the thorax (6.9%). For AIS3+ injuries, male cyclists < 60YO most often suffer head injuries (65.3%) followed by lower extremity injuries (17.9%) and injuries to the thorax (11.7%). Analyzing significant differences revealed significant differences between females and males for AIS2+ as well as for AIS3+ injuries with regard to injured body regions for cyclists < 60YO. Males have significantly higher odds of sustaining AIS2+ injuries to the thorax (OR = 0.54, *p*-value < 0.001), injuries to the abdomen (OR = 0.76, *p*-value = 0.028) and injuries to the upper extremities (OR = 0.58, *p*-value < 0.001). The odds for females suffering AIS2+ injuries to the spine (OR = 1.26, *p*-value = 0.002) and injuries to the lower extremities (OR = 1.56, *p*-value < 0.001), on the other hand, are significantly higher. For AIS3+ injuries, it was observed that females have significantly higher odds of suffering head injuries (OR = 1.29, *p*-value = 0.001) while males have significantly higher odds of suffering thorax injuries (OR = 0.56, *p*-value < 0.001).

For female cyclists ≥ 60YO, the three most commonly injured AIS2+ body regions are the lower extremities (39.8%) followed by the head (27.6%) and the upper extremities (12.7%). For male cyclists ≥ 60YO, the three most commonly injured AIS2+ body regions are the head (33.3%) followed by the lower extremities (25.5%) and the thorax (17.7%). For AIS3+ injuries we have observed the following order for females: head (59.7%) followed by lower extremities (29.3%) and thorax (7.6%). For AIS3+ injuries we have observed the following order for males: head (62.3%) followed by lower extremities (21.4%) and thorax (11.3%). Analyzing significant differences revealed significant differences between females and males AIS2+ as well as AIS3+ injuries with regard to injured body regions for cyclists ≥ 60YO. Males have significantly higher odds of sustaining AIS2+ injuries to the head (OR = 0.77, *p*-value < 0.001), injuries to the thorax (OR = 0.54, p- value < 0.001) and injuries to the spine (OR = 0.79, *p*-value = 0.014). The odds of females sustaining AIS2+ injuries to the lower extremities (OR = 1.93, *p*-value < 0.001), on the other hand, are significantly higher. For AIS3+ injuries it was observed that females have significantly higher odds of sustaining lower extremity injuries (OR = 1.52, p- value < 0.001) while males have significantly higher odds of sustaining thorax injuries (OR = 0.65, *p*-value = 0.004) and lower extremity injuries (OR = 1, *p*-value < 0.001).

#### Swedish Accident Data

In Supplementary Figure 3, the injured body regions as a function of sex and injury severity in Sweden are displayed. The *p*-values and OR of all body regions are summarized in Table 6.

**TABLE 6:** Share of injured body regions for AIS2+ and AIS3+ injuries, OR and p-value in Sweden for pedestrians and cyclists < 60YO and ≥ 60YO (*p-value < 5%).

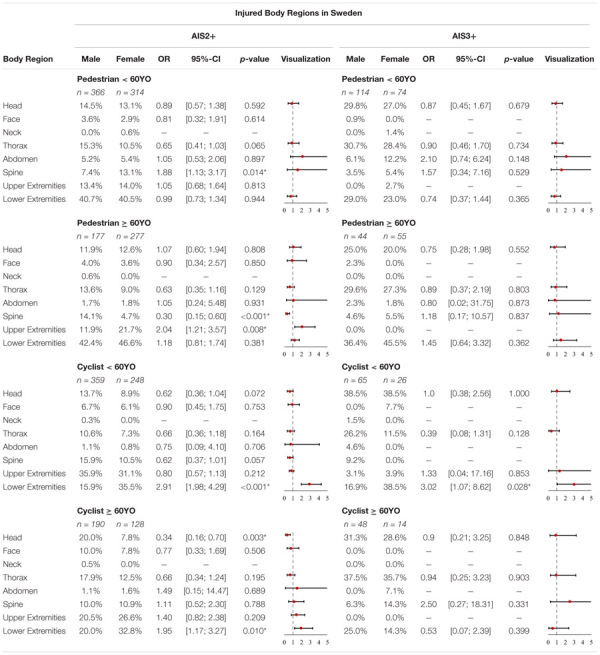

By analyzing the AIS2+ injuries the most commonly injured body regions for female pedestrians < 60YO are the lower extremities (40.4%) followed by the upper extremities (14%), the head (13.1%) and the spine (13.1%). For male pedestrians < 60YO the three most commonly injured AIS2+ body regions are the lower extremities (40.7%) followed by the thorax (15.3%) and the head (14.5%). These statistics change when considering more severe AIS3+ injuries. Here female pedestrians < 60YO most often suffer thorax injuries (28.4%) followed by head injuries (27%) and injuries to the lower extremities (23%). For AIS3+ injuries male pedestrians < 60YO most often suffer thorax injuries (30.7%) followed by head injuries (29.8%) and injuries to the lower extremities (28.8%). By analyzing if there are significant differences between females and males with regard to injured body regions, it can be seen that the odds for females suffering AIS2+ spine injuries, are significantly higher (OR = 1.88, *p*-value = 0.014).

For female pedestrians ≥ 60YO the three most commonly injured AIS2+ body regions are the lower extremities (46.6%) followed by the upper extremities (21.7%) and the head (12.6%). For male pedestrians ≥ 60YO the three most commonly injured AIS2+ body regions are the lower extremities (42.6%) followed by the spine (14.1%) and the thorax (13.6%). For AIS3+ injuries we have observed the following order for females ≥ 60YO: lower extremities (45.5%) followed by thorax (27.3%) and the head (20%). For AIS3+ injuries we have observed the following order for males ≥ 60YO: lower extremities (36.4%) followed by thorax (29.5%) and the head (25%). Analyzing significant differences revealed certain differences between females and males for AIS2+ with regard to injured body regions for pedestrians ≥ 60YO. Males, for example, have significantly higher odds (OR = 0.3, *p*-value < 0.001) of sustaining AIS2+ spine injuries while the odds for females sustaining upper extremity injuries are higher (OR = 2.04, *p*-value = 0.008).

Analyzing the AIS2+ injuries the three most commonly injured body regions for female cyclists < 60YO are the lower extremities (35.5%) followed by the upper extremities (31%) and the spine (10.5%). For male cyclists < 60YO the most commonly injured AIS2+ body regions are the upper extremities (35.9%) followed by the lower extremities (15.9%), the spine (15.9%) and the head (13.6%). These statistics change when considering more severe AIS3+ injuries. Here female cyclists < 60YO most often suffer lower extremity injuries (38.5%) followed by head injuries (38.5%) and injuries to the thorax (11.5%). For AIS3+ injuries male cyclists < 60YO most often suffer head injuries (38.5%) followed by thorax injuries (26.2%) and injuries to the lower extremities (16.9%). Analyzing significant differences revealed significant differences between males and females for AIS2+ as well as AIS3+ injuries of the lower extremities, with regard to injured body regions for cyclists < 60YO. Females have significantly higher odds of suffering AIS2+ (OR = 2.91, *p*-value < 0.001) or AIS3+ (OR = 3.02, *p*-value = 0.028) injuries of the lower extremities. For other body regions, no significant differences were observed.

For female cyclists ≥ 60YO the three most commonly injured AIS2+ body regions are the lower extremities (32.8%) followed by the upper extremities (26.6%) and the thorax (12.5%). For male cyclists ≥ 60YO the most commonly injured AIS2+ body regions are the upper extremities (20.5%) followed by the lower extremities (20%), head (20%) and the thorax (17.9%). For AIS3+ injuries we have observed the following order for females: thorax (35.7%) followed by head (28.6%), lower extremities (14.3%) and the spine (14.3%), respectively. For AIS3+ injuries we have observed the following order for males: thorax (35.7%) followed by head (31.2%) and lower extremities (25%). Analyzing significant differences revealed certain differences between females and males for AIS2+ with regard to injured body regions for cyclists ≥ 60YO. Males for example have significantly higher odds (OR = 0.34, *p*-value = 0.003) of sustaining AIS2+ head injuries while the odds for females sustaining lower extremity injuries is significantly higher (OR = 1.95, *p*-value = 0.01). For other body regions or AIS3+ injuries, no significant differences were observed.

### Detailed Injury Analyses for Significant AIS2+ and AIS3+ Injuries

#### Austrian Accident Data

Significant differences were only identified for pedestrian-to-passenger car collisions involving males and females in the Austrian accident data, shown in Table 7. Male pedestrians < 60YO have significantly higher odds of sustaining AIS2+ skull injuries (OR = 0.5, *p*-value = 0.008) while the odds for female < 60YO of sustaining AIS2+ lumbar spine injuries (OR = 7.89, *p*-value < 0.001), AIS2+ femur injuries (OR = 3.63, *p*-value = 0.042) and AIS2+ pelvic injuries (OR = 3.13, *p*-value < 0.001) are significantly higher.

**TABLE 7:** Share of AIS2+ and AIS3+ injuries with significant differences, OR and *p*-value in Austria for pedestrians and cyclists < 60YO and ≥ 60YO.

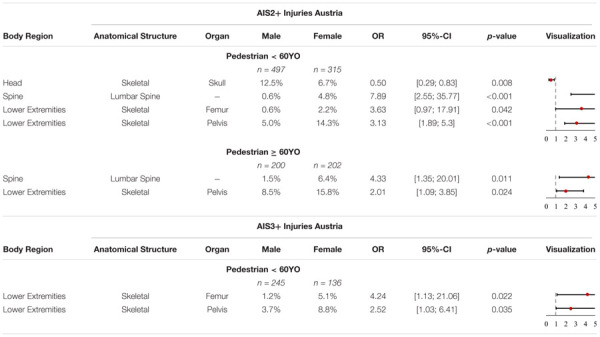

Significant differences were only seen for female pedestrians ≥ 60YO, whereby the odds for females sustaining AIS2+ lumbar spine injuries (OR = 4.33, *p*-value = 0.011) and AIS2+ pelvic injuries (OR = 2.01, *p*-value = 0.024) were observed to be significantly higher.

It was also observed that female pedestrians < 60YO have significantly higher odds of sustaining AIS2+ femur (OR = 4.24, *p*-value = 0.022) and AIS2+ pelvic (OR = 2.52, *p*-value = 0.035) injuries. For all other groups no significant differences were observed with regard to injuries sustained by males and females.

A summary of all these findings can be seen in Table 7.

#### Dutch Accident Data

Significant differences were observed for AIS2+ injuries, in terms of frequencies between males and females, in the Dutch data, all listed in Table 8. It can be observed that pedestrian < 60YO females have significantly higher odds of sustaining skeletal injuries of the thorax and the lower extremities. Skeletal injuries are always related to a fracture of a specific bone. Hence, the odds of females sustaining different fractures, i.e., the pelvis (OR = 1.90, *p*-value = 0.021) are significantly higher. Male pedestrians < 60YO in the Netherlands have significantly higher odds of sustaining AIS2+ concussive injuries (OR = 0.75, *p*-value = 0.041), AIS2+ spleen injuries (OR = 0.52, *p*-value = 0.045) and AIS2+ cervical spine injuries (OR = 0.66, *p*-value = 0.042). For pedestrians ≥ 60YO, it can be observed that the odds for females and males sustaining skeletal injuries to different body parts is significantly higher. Thus, the odds of females sustaining AIS2+ hand (OR = 2.13, *p*-value = 0.04), femur (OR = 1.35, *p*-value = 0.047) and tibia (OR = 1.3, *p*-value = 0.024) injuries, are significantly higher. Males, on the other hand, have significantly higher odds of sustaining rib cage (OR = 0.68, *p*-value = 0.046) and scapula (OR = 0.43, *p*-value = 0.017) injuries.

**TABLE 8:** Share of AIS2+ and AIS3+ injuries with significant differences, OR and *p*-value in the Netherlands for pedestrians and cyclists < 60YO and ≥ 60YO.

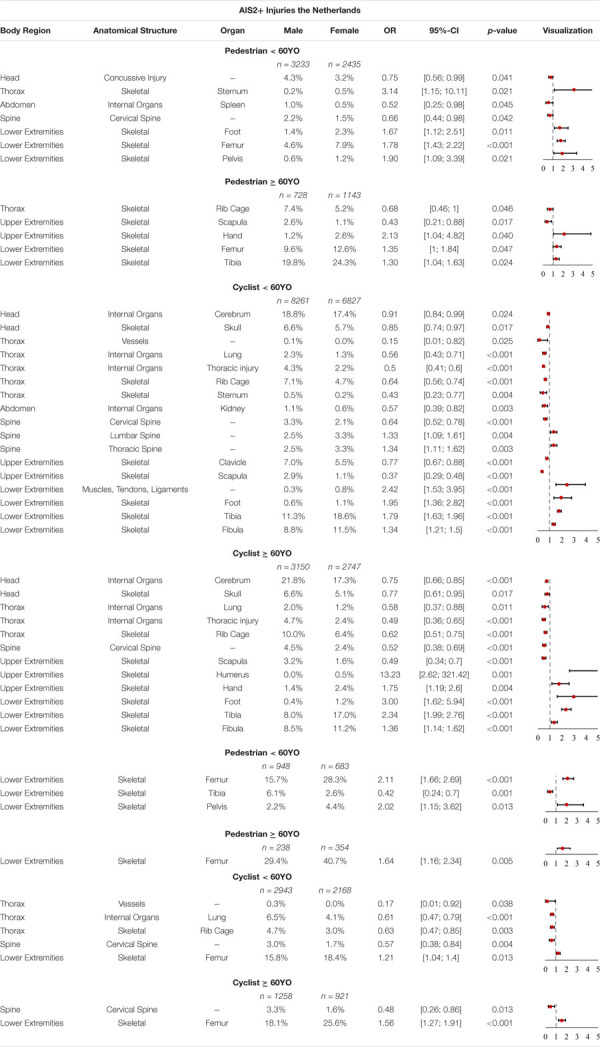

A significant difference was observed between males and females for a large number of AIS2+ injuries sustained by cyclists in cyclist-to-passenger car accidents. Female cyclists < 60YO have significantly higher odds of sustaining different spine and lower extremity injuries. The odds for male cyclists < 60YO, on the other hand, of sustaining different AIS2+ head, thorax, abdomen and cervical spine injuries, as well as skeletal injuries of the upper extremities, are higher. A similar picture can be seen for injuries sustained by cyclists ≥ 60YO. In this group, females have significantly higher odds of sustaining different skeletal injuries to the lower extremities, however, the odds are also higher for AIS2+ hand (OR = 1.75, *p*-value = 0.004) and AIS2+ humerus (OR = 13.23, *p*-value = 0.001) injuries. Again, males have significantly higher odds of sustaining different AIS2+ head, thorax, and cervical spine injuries, as well as skeletal injuries of the scapula.

The odds for female pedestrians and cyclists sustaining AIS3+ skeletal femur injuries are significantly higher than for males in the Dutch accident data, irrespective of age. Moreover, younger female pedestrians (<60YO) have significantly higher odds of sustaining AIS3+ pelvic injuries (OR = 2.02, *p*-value = 0.013). The odds for younger male pedestrians of sustaining AIS3+ skeletal tibia injuries (OR = 0.42, *p*-value = 0.001) are significantly higher. For younger male cyclists we observed that they have significantly higher odds of sustaining different types of thorax and cortical spine injuries. Furthermore, the odds of elderly male cyclists sustaining AIS3+ cortical spine injuries (OR = 0.48, *p*-value = 0.013) are also higher.

A summary of all these findings can be seen in Table 8.

#### Swedish Accident Data

Some significant differences were observed for AIS2+ injuries in the Swedish data for females and males, shown in Table 9. The odds of the group of pedestrian < 60YO males of sustaining AIS2+ lung injuries (OR = 0.47, *p*-value = 0.025) and AIS2+ thoracic injuries (OR = 0.39, *p*-value = 0.014) are significantly higher. Thoracic injuries include, among others, hemothorax, pneumothorax and hemopneumothorax. On the other hand, the odds for females < 60YO of sustaining different fractures (skeletal injuries) are higher. For the upper extremities, females have significantly higher odds of sustaining AIS2+ radius injuries (OR = 2.82, *p*-value = 0.003) while the odds for lower extremity injuries, sustaining AIS2+ pelvic injuries (OR = 2.04, *p*-value = 0.005) are significantly higher.

**TABLE 9:** Share of AIS2+ and AIS3+ injuries with significant differences, OR and *p*-value in Sweden for pedestrians and cyclists < 60YO and ≥ 60YO.

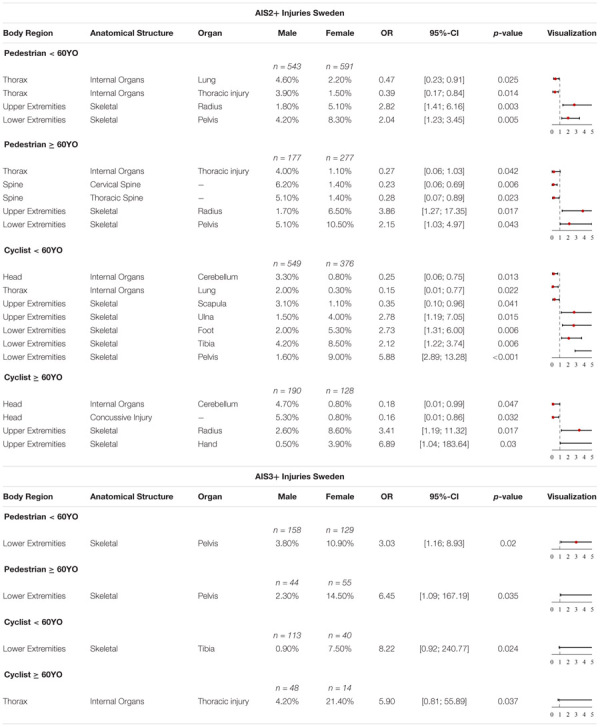

The group of pedestrian ≥ 60YO males have significantly higher odds of sustaining AIS2+ thoracic injuries (OR = 0.27, *p*-value = 0.042), AIS2+ injuries of the cervical spine (OR = 0.23, *p*-value = 0.006) and AIS2+ of the thoracic spine (OR = 0.28, *p*-value = 0.023). A similar trend can be seen for females for the group of ≥ 60YO as for < 60YO pedestrians. The odds for elderly females are also significantly higher for sustaining AIS2+ radius injuries (OR = 3.86, *p*-value = 0.017) and AIS2+ pelvic injuries (OR = 2.15, *p*-value = 0.043).

Significant differences were also observed in the Swedish accident data for male and female cyclists in terms of sustained injuries. Males < 60YO have significantly higher odds of sustaining AIS2+ cerebellum injuries (OR = 0.25, *p*-value = 0.013), AIS2+ lung injuries (OR = 0.23, *p*-value = 0.006) and AIS2+ skeletal injuries of the scapula (OR = 0.35, *p*-value = 0.041). The odds of females sustaining AIS2+ ulna injuries (OR = 2.78, *p*-value = 0.015), AIS2+ foot injuries (OR = 2.73, *p*-value = 0.006), AIS2+ tibia injuries (OR = 2.12, *p*-value = 0.006) and AIS2+ pelvic injuries (OR = 5.88, *p*-value < 0.001) are significantly higher. All injuries with observed significant differences for females involve skeletal injuries and thus fractures.

For elderly cyclists (≥60YO), males have significantly higher odds of sustaining AIS2+ head injuries. Furthermore, males also have significantly higher odds of sustaining AIS2+ cerebellum injuries (OR = 0.18, *p*-value = 0.047) and AIS2+ concussive injuries (OR = 0.16, *p*-value = 0.032). Again, females displayed significantly higher odds of suffering skeletal injuries. They also have significantly higher odds of sustaining AIS2+ radius injuries (OR = 3.41, *p*-value = 0.017) and AIS2+ skeletal injuries of the hands (OR = 6.89, *p*-value = 0.003).

For AIS3+ injuries, significant differences in injuries sustained by females were observed in the Swedish accident data. Hence, the odds for young female pedestrians (<60YO) and elderly female pedestrians (≥60YO) of sustaining AIS3+ pelvic injuries (OR = 3.03, *p*-value = 0.02 and OR = 6.45, *p*-value = 0.035, respectively) are significantly higher. Young female cyclists on the other hand have significantly higher odds of sustaining AIS3+ tibia injuries (OR = 8.22, *p*-value = 0.024) while elderly female cyclists are at a higher risk of suffering thoracic injuries (OR = 5.9, *p*-value = 0.037).

A summary of all these findings can be seen in Table 9.

### Injury Severity

With regard to this section, please refer to the Supplementary Table 13 for further evaluation of significant differences between younger (<60YO) and older (≥60YO) pedestrian and cyclists identified in all three accident datasets.

The odds ratios and *p*-values based on the Hypothesis Tests are summarized for all three data samples in Table 10 and Supplementary Table 13.

**TABLE 10:** Injury Severity, OR and *p*-value for pedestrians and cyclists < 60YO and ≥ 60YO in Austrian, Dutch, and Swedish accident data (*p-value < 5%).

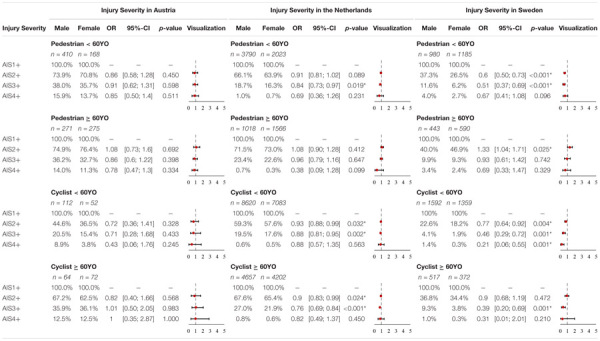

#### Austrian Accident Data

In the Austrian dataset, no significant differences in injury severity were identified for pedestrian-to-passenger car, as well as cyclist-to-passenger car collisions, in both age groups for females and males.

No significant differences were observed on analyzing if age group has influence on injuries sustained by pedestrians. A slightly different trend can be seen for cyclist-to-passenger car collisions. Older cyclists (≥60YO) have significantly higher odds of sustaining AIS2+ (OR = 0.4, *p*-value < 0.001) and AIS3+ (OR = 0.42, *p*-value = 0.001) injuries than younger cyclists (<60YO).

#### Dutch Accident Data

The odds for younger males (<60YO) in the Netherlands, involved in pedestrian-to-passenger car collisions of sustaining AIS2+ (OR = 0.84, *p*-value = 0.019) injuries, were significantly higher than for younger females. No significant differences were identified in injury severity between male and female pedestrians ≥ 60YO.

A slightly different trend can be seen for cyclist-to-passenger car collisions. Males in the cyclist < 60YO group have significantly higher odds of sustaining AIS2+ (OR = 0.93, *p*-value = 0.032) and AIS3+ (OR = 0.88, *p*-value = 0.002) injuries than females. Elderly males also have significantly higher odds of sustaining AIS2+ (OR = 0.9, *p*-value = 0.024) and AIS3+ (OR = 0.76, *p*-value < 0.001) injuries than females.

Significant differences were observed on analyzing if age group has influence on injuries sustained by pedestrians. Older pedestrians (≥60YO) have significantly higher odds of sustaining AIS2+ (OR = 0.72, *p*-value < 0.001) and AIS3+ (OR = 0.73, *p*-value < 0.001) injuries than younger pedestrians (<60YO). A similar trend can be seen for cyclist-to-passenger car collisions. Older cyclists (≥60YO) have significantly higher odds of sustaining AIS2+ (OR = 0.71, *p*-value < 0.001) and AIS3+ (OR = 0.7, *p*-value < 0.001) injuries than younger cyclists (<60YO).

#### Swedish Accident Data

Significant differences were observed between males and females in the group of pedestrians < 60YO. Males have significantly higher odds of sustaining AIS2+ (OR = 0.6, *p*-value < 0.001) and AIS3+ (OR = 0.51, *p*-value < 0.001) injuries.

The odds for pedestrian ≥ 60YO females of sustaining AIS2+ (OR = 1.33, *p*-value = 0.025) injuries were significantly higher.

Significant differences were again observed for male and female cyclist injuries in Sweden. The odds for males < 60YO of sustaining AIS2+ (OR = 0.77, *p*-value = 0.004), AIS3+ (OR = 0.46, *p*-value = 0.001) and AIS4+ (OR = 0.21, *p*-value = 0.001) injuries were observed to be significantly higher.

For the group of cyclists ≥ 60YO in Sweden, the odds for male cyclists ≥ 60YO of sustaining AIS3+ injuries are significantly higher (OR = 0.39, *p*-value = 0.001) when involved in a cyclist-to-passenger car collision.

Significant differences were observed on analyzing if age group has influence on injuries sustained by. Older pedestrians (≥60YO) have significantly higher odds of sustaining AIS2+ (OR = 0.58, *p*-value < 0.001) injuries than younger pedestrian (<60YO). A similar trend can be seen for cyclist-to-passenger car collisions. Older cyclists (≥60YO) have significantly higher odds of sustaining AIS2+ (OR = 0.47, *p*-value < 0.001) and AIS3+ (OR = 0.42, *p*-value < 0.001) injuries than younger cyclists (<60YO).

### In-Depth Analysis of Accident Data

With regard to this section, please refer to the Supplementary Material for further evaluation of significant differences between females and males identified in the Austrian accident data.

**FIGURE 1:**
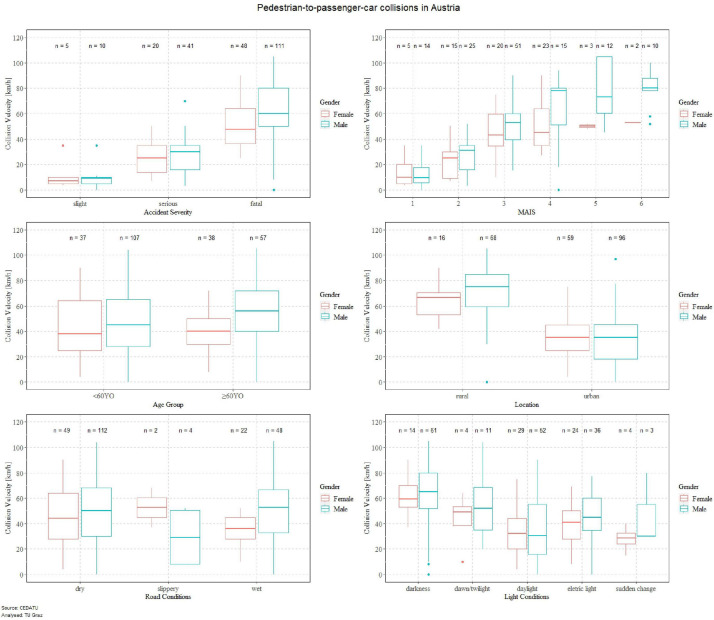
In-depth analysis of Austrian accident data for pedestrian-to-passenger car collisions.

For the in-depth analysis of the Austrian accident data for pedestrians involved in pedestrian-to-passenger car collisions (Figure 1), it can be seen that for almost all parameters, the mean collision velocities for males are higher than for females. Moreover, it can be seen that higher injury severities are also related to higher collision velocities. Analyzing if there are significant differences in accident severity for females and males, it was found that elderly male pedestrians (≥60YO) have significantly higher odds of sustaining fatal injuries (Supplementary Table 15). The trend of higher collision velocities for males can also be seen for the maximum abbreviated injury scale (MAIS) level. By analyzing if there are significant differences in MAIS levels for females and males, it was found that younger female pedestrians (<60YO) have significantly higher odds of sustaining MAIS4 injuries (Supplementary Table 16). The mean collision speed for elderly pedestrians (≥60YO) is slightly higher in the data sample than for younger pedestrians (<60YO) for both females and males. For accidents occurring in rural areas, it is noticeable that the collision speed is higher than in urban areas. Also, for accident location, significant differences were observed between males and females (Supplementary Table 14). The odds of elderly male pedestrians (≥60YO) being involved in an accident in rural areas were observed to be higher. As pedestrian accident location was also included in the Dutch and Swedish accident data, significant differences were observed for that region too. The odds for male pedestrians in the Netherlands and in Sweden being involved in an accident in rural areas, irrespective of age, were significantly higher. Collision speed in Austrian accident data for males in both dry and wet road conditions were higher than for females. The opposite trend was observed for slippery road surfaces, however, only a few cases had been reported. On analyzing if there are significant differences for females and males with regard to road conditions, none were found in the Austrian and Dutch accident data (Supplementary Table 17). Accidents at night-time (electric light or darkness) occurred at higher collision speeds than accidents in daylight. Moreover, the likelihood of males being involved in an accident in darkness was higher than for females in Austria and the Netherlands (Supplementary Table 18). Analyzing the influence of alcohol on pedestrian-to-passenger-car collisions, it was found that the odds of pedestrian males being intoxicated by alcohol were higher than females (Supplementary Table 19).

**FIGURE 2:**
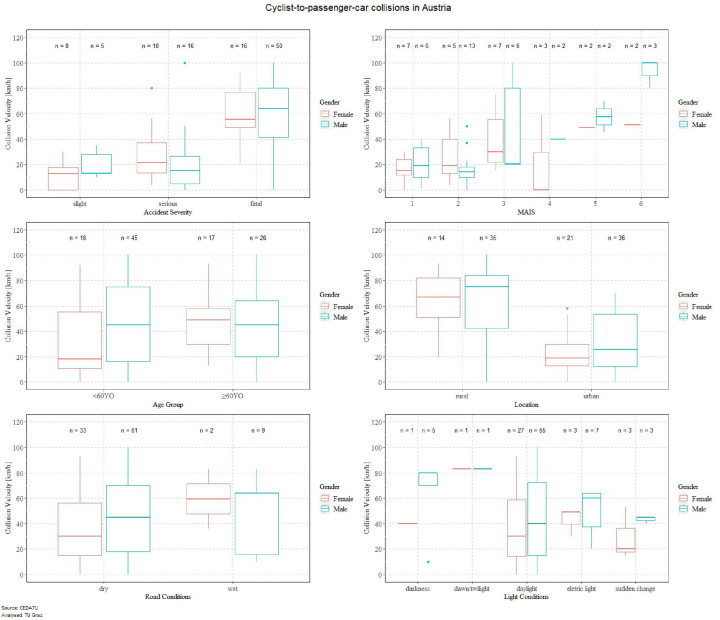
In-depth analysis of Austrian accident data for cyclist-to-passenger car collisions.

For the in-depth analysis of cyclists involved in pedestrian-to-passenger car collisions (Figure 2) included in the Austrian accident data, it can also be seen that for a considerable number of parameters the mean collision velocity for males is faster than for females. It can also be seen that higher injury severities are related to higher collision velocities. On analyzing if there are significant differences in accident severity for females and males, it was found that the risk of sustaining fatal injuries is significantly higher for male cyclists, irrespective of age (Supplementary Table 15). The trend of higher collision velocities for males can also be seen for most of the MAIS levels. Analyzing significant differences in MAIS level for females and males, it was found that younger female cyclists (<60YO) have significantly higher odds of sustaining MAIS3 injuries (Supplementary Table 16). The mean collision speed for elderly female cyclists (≥60YO) is slightly higher in the data sample than for younger female cyclists (<60YO). However, age was not found to influence speed for males. For accidents that had occurred in rural areas, it was noticeable that the collision speeds were higher than in urban areas. Accident location was not observed to make any significant difference between males and females in the Austrian data. As cyclist accident location was also included in the Dutch and Swedish accident data, significant differences could also be observed for those regions. Male cyclists in the Netherlands and Sweden have significantly higher odds of being involved in an accident in rural areas, irrespective of age (Supplementary Table 14). Collision speed in the Austrian accident data was found to be higher for males than females in dry road conditions. The opposite trend was observed for wet road conditions, however, only a few cases had been reported. For all road conditions no significant differences between males and females can be observed in Austrian and Dutch accident data (Supplementary Table 17). Most of the cyclist accidents involving passenger cars had occurred during daylight, although the collision speed was higher also for cyclist accidents at night-time (electric light or darkness). Furthermore, light conditions were not observed to have made any significant difference between males and females in Austrian accident data. In Dutch accident data it was observed that the likelihood of males being involved in an accident in darkness was higher than for females (Supplementary Table 18). The influence of alcohol was observed for cyclists in the Austrian accident data (Supplementary Table 19).

## Discussion

The accident databases show several significant differences due to the applied data sources and their original purpose.

Although some authors have previously tried to combine data from different databases, or extrapolate from one country to others (Kreiss et al., 2015), this was not done in the current study. Instead, trends from the different data sources have been compared and the advantages of each of the data sets were utilized. The Dutch dataset showed the highest number of cases, the Swedish dataset is the only dataset that covers all cases from one country, and is therefore most representative and unbiased. The Austrian dataset had the highest level of detail and therefore allowed the authors to perform additional analyses. It would be beneficial to have a representative, European-wide, long-term in-depth database to eliminate the limitations mentioned above.

The data in this study was collected during different time periods. However, when analyzing the Dutch data, no significant change in injuries over the years was observed, hence it has been assumed by the authors that this parameter does not influence the results. The data selection criteria, i.e., being recorded by both police and hospital or involvement of at least one vehicle, was made in order to obtain as comparable data between the countries as possible. Using this criteria is necessary, due to a significant difference in hospital and police reported data having been observed in previous studies (Juhra et al., 2012). As a consequence of using only matched police and hospital reported accidents, the data do not cover all accidents. For example, only 30% of all cases in the STRADA database are reported by both police and hospital (Yamazaki, 2018). On the other hand, as the present study includes accidents involving passenger cars, there should be a higher inclusion of the total number of crashes as the police are more likely to have reported an accident involving a motor vehicle.

For the Austrian data, a shift toward serious and fatal accidents can be seen when comparing the CEDATU database with the national statistics (Supplementary Figure 6). This is because the original focus of the CEDATU database was to collect data on reconstructed fatal accidents in Austria (Tomasch and Steffan, 2006; Tomasch et al., 2008). In recent years, increasingly accidents involving minor as well as severe injuries have been included in the database.

Similarly, a shift toward more severe injuries and fatal accidents can be seen in the Dutch dataset. The Dutch police register contains 90% of all fatal road accidents, although unfortunately it is less comprehensive for accidents of lesser severity (Reurings and Stipdonk, 2011).

Despite the shift toward more severe injuries and fatal accidents in the Austrian and Dutch datasets in comparison to national statistics, the datasets have been very beneficial when it comes to comparisons of injuries sustained by females and males relative to each other. For the analysis of the most relevant AIS2+ body regions, one should on the other hand mainly rely on the results based on the STRADA database.

A summary with all accident parameters and injuries showing significant differences between males and females can be seen in Tables 11, 12. For more details on the exact values for OR and *p*-value have a look on the result section and the Supplementary Material.

**TABLE 11:** Summary of all significant differences for different body regions and accident parameters for pedestrians and cyclists for Austria (AUT), Netherlands (NL), and Sweden (SWE).

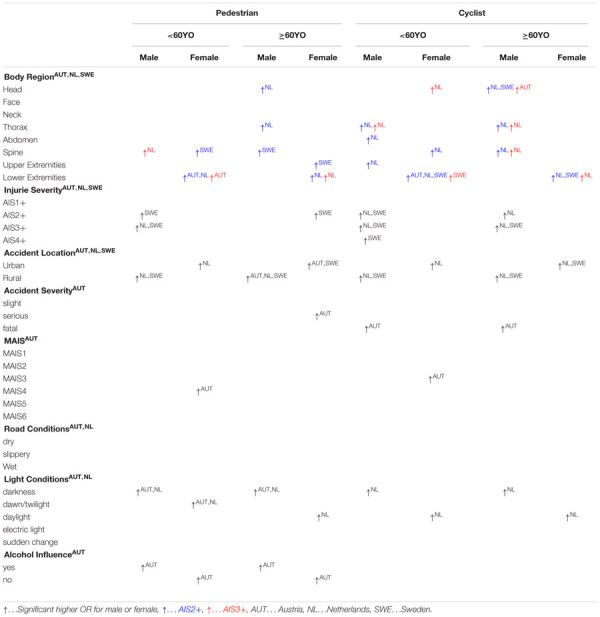

**TABLE 12:** Summary of all single injuries with significant differences for pedestrians and cyclists for Austria (AUT), Netherlands (NL), and Sweden (SWE).

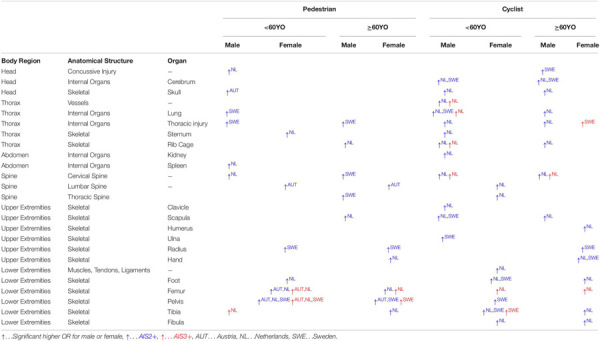

### Body Regions

The current analysis shows that the body regions head, thorax, upper extremities and lower extremities are more or less equally relevant for pedestrian and cyclist statistics when it comes to injury mitigation. Only a small difference was seen in the different databases, in that the order may differ between the most relevant body regions. These findings are in line with other studies which have also identified these body regions as most commonly injured by pedestrians and cyclists involved in passenger car collisions (Otte et al., 2012; Weijermars et al., 2016; Wisch et al., 2017; Saadé et al., 2020).

Predominant in the databases and groups, the head was the most frequently injured body region. The fact that the head is one of the most relevant body region when it comes to injury mitigation for pedestrian and cyclist accidents could be explained by the fact that the head is one of the most vulnerable body region. This can also be seen when having a look into the AIS Codebook (AAAM, 2005) where the majority of injuries related to the head are coded as AIS2+. Head injuries were less frequent for cyclists in the Swedish dataset. This might be a result of high helmet wearing rates (Otte et al., 2015; Leo et al., 2019a).

Another fact that can be seen through all databases is that females often have significantly higher odds of sustaining injuries to the lower extremities. This was observed for pedestrians as well as for cyclists. For cyclists, this may can be explained in possible differences in the type of bicycle they ride, their riding speed and the type of accidents they are involved in Boele-Vos et al. (2017), Fyhri et al. (2019), Prati et al. (2019). Male cyclists for example more often ride on racing bikes whereas (elderly) female cyclists more often ride on pedelecs (electrically assisted bicycles) (Boele-Vos et al., 2017). Riding on a racing bike is related to higher riding speeds, a different seating position and the type of accidents may also be different. This could explain differences in injuries sustained. In addition, osteoporosis is much more common in women than in men (Alswat, 2017), which may also explain more fractures in women, such as femur/hip/pelvic fractures.

The proportion of AIS2+ injuries to the upper extremities is rather considerable, especially in Swedish accident data. However, looking at AIS3+ injuries, injuries of the upper extremities are not particularly common. Nevertheless, assessing long-term consequences of injuries, it has been shown that 85% of AIS3 upper extremity injuries, result in permanent medical impairment (Malm et al., 2008). In contrast, while thorax injuries were common for AIS 3+ injuries in all three databases, these injuries rarely result in permanent medical impairment. This illustrates that when taking long-term consequences of injuries into account, preventive measures must target upper extremity injuries as well. One last fact is that significant differences for the frequency of spinal injuries between males and females were observed in the current study. However, the trend was not consistent within the different age groups.

### Detailed Injuries

For the detailed injury types, significant differences in injuries sustained by females and males were identified in all three databases. This information is very valuable with regard to the development and improvement of HBMs for virtual testing. Knowing which injuries are most common, and for which injuries significant differences can be seen between males and females, is necessary to specify what must be predicted by the HBMs.

For lower extremities (incl. pelvis), it was found that females have significantly higher odds of sustaining skeletal injuries. In all three data sets, female pedestrians showed higher odds of sustaining pelvic injuries than males. This is in line with a study by Starnes et al. (2011) and Klug et al. (2015). Starnes also concluded that males are significantly more likely to suffer tibia fractures. This, however, cannot be confirmed in the present study, due to different results being observed for tibia fractures in the different databases.

Furthermore, female cyclists showed significantly higher odds than males for tibia fractures in the Swedish and the Dutch dataset. This finding may be influenced by the fact that females and males ride on different types of bicycle frames, producing a different interaction with the lower extremities.

### Injury Severity and Exposure

When comparing injury severity between males and females, males have significantly higher odds of sustaining more severe injuries compared to females in pedestrian and cyclist accidents. This is especially the case when looking at the Swedish data. From the Austrian database, which includes information on collision speeds of passenger cars, it can be seen that the collision speeds of passenger cars were higher in collisions involving males compared with females. A similar trend can also be seen when looking at cyclist-to-passenger car collisions in Austria.

The Austrian, Dutch and Swedish data shows that the odds of females being involved in a rural accident are lower than for males. It was also shown that males are more likely involved in accidents during nights. The analysis of the Austrian data has shown that these types of accidents are related to higher collision speeds. Hence, the observation of higher injury severity among males is more likely a function of the exposure to higher collision speeds of passenger cars rather than a question of the sex. In the future, it should be analyzed if any significant differences in injury severities are apparent at similar energy levels of the accidents. However, this requires additional crash data unavailable in the current datasets. Applying additional filters narrows down our numbers too much, so that no meaningful analyses can be done. Further investigations should be done to study gender-specific differences, which might lead to different accident scenarios. Some first indications have been observed in this study, showing that the types of accidents where females (more likely to be injured during daytime, inner-city) are severely injured might differ from males (higher odds to be injured during night-time at rural roads in an alcoholized state).

Regarding the age of the vulnerable road users, the Dutch and Swedish data shows that older (≥60YO) pedestrian and cyclist have significant higher odds of sustaining AIS2+ injuries. A similar trend can also be seen for AIS3+ injuries. The Austrian accident data show significant higher odds for elderly (≥60YO) cyclists sustaining AIS2+ and AIS3+ injuries as well. This is in line with previous studies which conclude that elderly pedestrians (≥60YO) tend to suffer more severe injuries than younger pedestrians (<60YO) (Davis, 2001; Niebuhr et al., 2016; Saadé et al., 2020).

### Outlook

Recent studies have shown that through the implementation of autonomous emergency breaking (AEB) systems, the collision velocities in pedestrian and cyclist-to-passenger car collisions will be drastically lowered (Gruber et al., 2019; Leo et al., 2020). Reducing the collision speed will also lead to a change in impact conditions, i.e., lower head impact velocities, Leo et al. (2020). Presumably, this fact will also lead to a shift in the injuries sustained by pedestrians and cyclists in the foreseeable future.

Once comparable FE Human Body Models of an average female and male are available, the isolated sex-specific differences in injury risk caused by differences in loadings due to differences in anthropometries and influences of individual factors such as age, and injury thresholds, could and should be investigated. Moreover, by means of virtual testing the diversity of accident scenarios and the human population can be addressed by including different pre-collision behavior.

### Conclusion

The conclusion of this study is that female and male pedestrians and cyclists have significant different odds of sustaining injuries in accidents involving passenger cars. This trend can be seen for injuries to different body regions, single injuries and also for injury severity. For example, the results show that the odds of sustaining skeletal injuries to the lower extremities (incl. pelvis) in females are significantly higher. Moreover, significant differences in injuries severity for younger (<60YO) and elderly (≥60YO) pedestrians and cyclists were observed. In-depth analyses of Austrian accident data have shown that collision velocities are higher for male pedestrians and cyclists than for females in passenger car collisions. Furthermore, it was observed in all datasets, that the odds of females being involved in a rural accident or an accident at night are lower than for males.

The findings of this study highlight the need for policy makers and stakeholders to work toward developing safety features and assessment tools (e.g., integrated assessment) that take into account population diversity of sex and age and other individual related factors.

## Data Availability Statement

The data analyzed in this study is subject to the following licenses/restrictions: Raw data from accident databases from Austria, Netherlands, and Sweden are not publicly available (due to data protection regulations). Requests to access these datasets should be directed to CL, christoph.leo@tugraz.at and CK, corina.klug@tugraz.at.

## Author Contributions

CL carried out the data analysis and manuscript preparation. CK designed the study and supervised the data analysis. MR, NB, RD, AL, ET, and CK provided comments, feedback, and edited the manuscript. MR, NB, RD, and ET extracted the data from the Dutch, Swedish and Austrian accident databases respectively. All authors read and approved the final manuscript.

## Conflict of Interest

The authors declare that the research was conducted in the absence of any commercial or financial relationships that could be construed as a potential conflict of interest.

## References

[B1] AAAM (2005). *Abbreviated Injury Scale 2005.* Des Plaines IL: AAAM.

[B2] Alswat K. A. (2017). Gender disparities in osteoporosis. *J. Clin. Med. Res.* 9 382–387. 10.14740/jocmr2970w 28392857 PMC5380170

[B3] Andrade C. (2015). Understanding relative risk, odds ratio, and related terms: as simple as it can get. *J. Clin. Psychiatry* 76 e857–e861. 10.4088/JCP.15f10150 26231012

[B4] Boele-Vos M. J. van Duijvenvoorde K. Doumen M. J. A. Duivenvoorden C. W. A. E. Louwerse W. J. R. Davidse R. J. (2017). Crashes involving cyclists aged 50 and over in the Netherlands: an in-depth study. *Accid. Anal. Prev.* 105 4–10. 10.1016/j.aap.2016.07.016 27544622

[B5] Bose D. Segui-Gomez M. Crandall J. R. (2011). Vulnerability of female drivers involved in motor vehicle crashes: an analysis of US population at risk. *Am. J. Public Health* 101 2368–2373. 10.2105/AJPH.2011.300275 22021321 PMC3222446

[B6] Davis G. (2001). Relating severity of pedestrian injury to impact speed in vehicle-pedestrian crashes: simple threshold model. *Transport. Res. Record* 1773 108–113. 10.3141/1773-13 12716185

[B7] Forman J. Poplin G. S. Shaw C. G. McMurry T. L. Schmidt K. Ash J. (2019). Automobile injury trends in the contemporary fleet: belted occupants in frontal collisions. *Traffic Inj. Prev.* 20 607–612. 10.1080/15389588.2019.1630825 31283362

[B8] Fyhri A. Johansson O. Bjørnskau T. (2019). Gender differences in accident risk with e-bikes—Survey data from Norway. *Accid. Anal. Prev.* 132:105248. 10.1016/j.aap.2019.07.024 31419619

[B9] Gruber M. Kolk H. Klug C. Tomasch E. Feist F. Schneider A. (2019). “The effect of P-AEB system parameters on the effectiveness for real world pedestrian accidents,” in *Proceedings of the 26th ESV Conference Proceedings*, ed. NHTSA (Washington, DC: NHTSA).

[B10] Howard C. Linder A. (2014). *Review of Swedish Experiences Concerning Analysis of People Injured in Traffic Accidents.* Brussels: Belgian Road Safety Institute

[B11] Juhra C. Wieskötter B. Chu K. Trost L. Weiss U. Messerschmidt M. (2012). Bicycle accidents - do we only see the tip of the iceberg? A prospective multi-centre study in a large German city combining medical and police data. *Injury* 43 2026–2034. 10.1016/j.injury.2011.10.016 22105099

[B12] Klug C. Weinberger M. Tomasch E. Feist F. Sinz W. Steffan H. (2015). “Pelvic and femoral injuries in car-to-pedestrian accidents,” in *Proceedings of the 2015 IRCOBI Conference Proceedings*, ed. International Research Council on the Biomechanics of Injury (Lyon: IRCOBI), 49–63.

[B13] Kreiss J. Feng G. Krampe J. Meyer M. Niebuhr T. Pastor C. (2015). “Extrapolation of GIDAS accident data to Europe,” in *Proceedings of the 24th ESV Conference Proceedings*, ed. NHTSA (Washington, DC: NHTSA).

[B14] Kullgren A. Krafft M. (2010). “Gender analysis on whiplash seat effectiveness: results from real-world crashes,” in *Proceedings of the 2010 IRCOBI Conference Proceedings*, Hanover, ed. International Research Council on the Biomechanics of Injury (Hanover: IRCOBI), 17–28.

[B15] Leo C. Gruber M. Feist F. Sinz W. Roth F. Klug C. (2020). “The effect of autonomous emergency braking systems on head impact conditions for pedestrian and cyclists in passenger car collisions,” in *Proceedings of the 2020 IRCOBI Conference Proceedings*, ed. International Research Council on the Biomechanics of Injury (Munich: IRCOBI), 330–357.

[B16] Leo C. Klug C. Ohlin M. Linder A. (2019b). “Analysis of pedestrian injuries in pedestrian-car collisions with focus on age and gender,” in *Proceedings of the 2019 IRCOBI Conference Proceedings*, ed. International Research Council on the Biomechanics of Injury (Florence: IRCOBI), 256–257.

[B17] Leo C. Klug C. Ohlin M. Bos N. Davidse R. Linder A. (2019a). Analysis of Swedish and Dutch accident data on cyclist injuries in cyclist-car collisions. *Traffic Inj. Prev.* 20 S160–S162. 10.1080/15389588.2019.1679551 31725328

[B18] Linder A. Svedberg W. (2019). Review of average sized male and female occupant models in European regulatory safety assessment tests and European laws: gaps and bridging suggestions. *Accid. Anal. Prev.* 127 156–162. 10.1016/j.aap.2019.02.030 30884388

[B19] Linder A. Svensson M. Y. (2019). Road safety: the average male as a norm in vehicle occupant crash safety assessment. *Interdiscip. Sci. Rev.* 44 140–153. 10.1080/03080188.2019.1603870

[B20] Linder A. Davidse R J Iraeus J. John J. D. Keller A. Klug C. (2020). “VIRTUAL - a European approach to foster the uptake of virtual testing in vehicle safety assessment,” in *Proceedings of the 8th Transport Research Arena*, Helsinki: TRA.

[B21] Malm S. Krafft M. Kullgren A. Ydenius A. Tingvall C. (2008). Risk of permanent medical impairment (RPMI) in road traffic accidents. *Ann. Adv. Automot. Med.* 52 93–100. 19026226 PMC3256772

[B22] Mattsson K. Ungerbäck A. (2013). *Vägtrafikolyckor: Handledning vid rapportering.* Borlänge: Transportstyrelsen.

[B23] McHugh M. (2009). The odds ratio: calculation, usage, and interpretation. *Biochem. Med.* 120–126. 10.11613/BM.2009.011

[B24] Mitchell Z. A. Cameron R. B. (2020). “Female vs. Male relative fatality risk in fatal crashes,” in *Proceedings of the 2020 IRCOBI Conference Proceedings*, ed. International Research Council on the Biomechanics of Injury (Munich: IRCOBI), 47–85.

[B25] National Board of Health and Welfare (2010). *International Statistical Classification of Diseases and Related Health Problems - Systematic listing: Swedish version 2011 (ICD-10-SE).* Stockholm: National Board of Health and Welfare

[B26] Niebuhr T. Junge M. Rosén E. (2016). Pedestrian injury risk and the effect of age. *Accid. Anal. Prev.* 86 121–128. 10.1016/j.aap.2015.10.026 26547018

[B27] Otte D. Jänsch M. Haasper C. (2012). Injury protection and accident causation parameters for vulnerable road users based on German In-Depth Accident Study GIDAS. *Accid. Anal. Prev.* 44 149–153. 10.1016/j.aap.2010.12.006 22062349

[B28] Otte D. Jänsch M. Morandi A. Orsi C. Stendardo A. Bogerd C. P. (2015). *Final Report of Working Group 1: In-Depth Accident Observations and Injury Statistics.* Brussels: COST Action TU1101 / HOPE

[B29] Pipkorn B. Iraeus J. Lindkvist M. Puthan P. Bunketorp O. (2020). Occupant injuries in light passenger vehicles-A NASS study to enable priorities for development of injury prediction capabilities of human body models. *Accid. Anal. Prev.* 138:105443. 10.1016/j.aap.2020.105443 32059123

[B30] Prati G. Fraboni F. de Angelis M. Pietrantoni L. (2019). Gender differences in cyclists’ crashes: an analysis of routinely recorded crash data. *Int. J. Inj. Contr. Saf. Promot.* 26 391–398. 10.1080/17457300.2019.1653930 31429363

[B31] Reurings M. C. B. Stipdonk H. L. (2011). Estimating the number of serious road injuries in the Netherlands. *Ann. Epidemiol.* 21 648–653. 10.1016/j.annepidem.2011.05.007 21820630

[B32] Saadé J. Cuny S. Labrousse M. Song E. Chauvel C. Chrétien P. (2020). “Pedestrian injuries and vehicles-related risk factors in car-to-pedestrian frontal collisions,” in *Proceedings of the 2020 IRCOBI Conference Proceedings*, ed. International Research Council on the Biomechanics of Injury (Munich: IRCOBI), 278–289.

[B33] Schneider L. W. (1983). *Development of Anthropometrically Based Design Specifications for an Advanced Adult Anthropomorphic Dummy Family, Final Report.* Report Number: UMTRI-83-53-1, Vol. 1 Michigan, MI: University of Michigan Transportation Research Institute.

[B34] Simms C. Wood D. (2009). *Pedestrian and Cyclist Impact: A Biomechanical Perspective.* Dordrecht: Springer Netherlands.

[B35] Starnes M. J. Hadjizacharia P. Chan L. S. Demetriades D. (2011). Automobile versus pedestrian injuries: does gender matter? *J. Emerg. Med.* 40 617–622. 10.1016/j.jemermed.2008.03.012 18842385

[B36] Swedish Government Offices (1965). *SFS 1965:561: last update in SFS 2014:1244, 2014.* Rosenbad: Swedish Government Offices

[B37] SWOV (2016). *Data Sources.* The Hague: SWOV.

[B38] Szumilas M. (2010). Explaining odds ratios. *J. Can. Acad. Child Adolesc. Psychiatry* 19 227–229. 20842279 PMC2938757

[B39] Tomasch E. Steffan H. (2006). “ZEDATU – zentrale datenbank tödlicher unfälle in österreich – a central database of fatalities in Austria,” in *Proceedings of the 2nd International Conference on ESAR “Expert Symposium on Accident Research”*, Hanover: ESAR.

[B40] Tomasch E. Steffan H. Darok M. (2008). “Retrospective accident investigation using information from court,” in *Proceedings of the Transport Research Arena Europe 2008 (TRA)*, Ljubljana: TRA.

[B41] Weijermars W. Bos N. Stipdonk H. L. (2016). Serious road injuries in The Netherlands dissected. *Traffic Inj. Prev.* 17 73–79. 10.1080/15389588.2015.1042577 26042645

[B42] Wisch M. Lerner M. Vukovic E. Hynd D. Fiorentino A. Fornells A. (2017). “Injury patterns of older car occupants, older pedestrians or cyclists in road traffic crashes with passenger cars in europe – results from SENIORS,” in *Proceedings of the 2017 IRCOBI Conference Proceedings*, ed. International Research Council on the Biomechanics of Injury (Antwerp: IRCOBI), 63–78.

[B43] World Health Organization (2018). *Global Status Report on Road Safety 2018.* Geneva: World Health Organization.

[B44] Yamazaki R. (2018). Strada Bortfallshandbok 2018 – Information om Täckning och Bortfall i Rapportering till Transportstyrelsens Vägolycksdatabas. Sweden: Swedish Transport Agency.

[B45] Zhang K. Cao L. Fanta A. Reed M. P. Neal M. O. Wang J.-T. (2017). An automated method to morph finite element whole-body human models with a wide range of stature and body shape for both men and women. *J. Biomech.* 60 253–260. 10.1016/j.jbiomech.2017.06.015 28668185

