# Supplementary material for: Are There Any Significant Differences in Terms of Age and Sex in Pedestrian and Cyclist Accidents?
Source: Front Bioeng Biotechnol. 2021 May 24;9:677952. doi: 10.3389/fbioe.2021.677952 (PMC8183819; doi:10.3389/fbioe.2021.677952)
Supplement: Supplementary file 1 [file Data_Sheet_1.PDF]

Supplementary Material

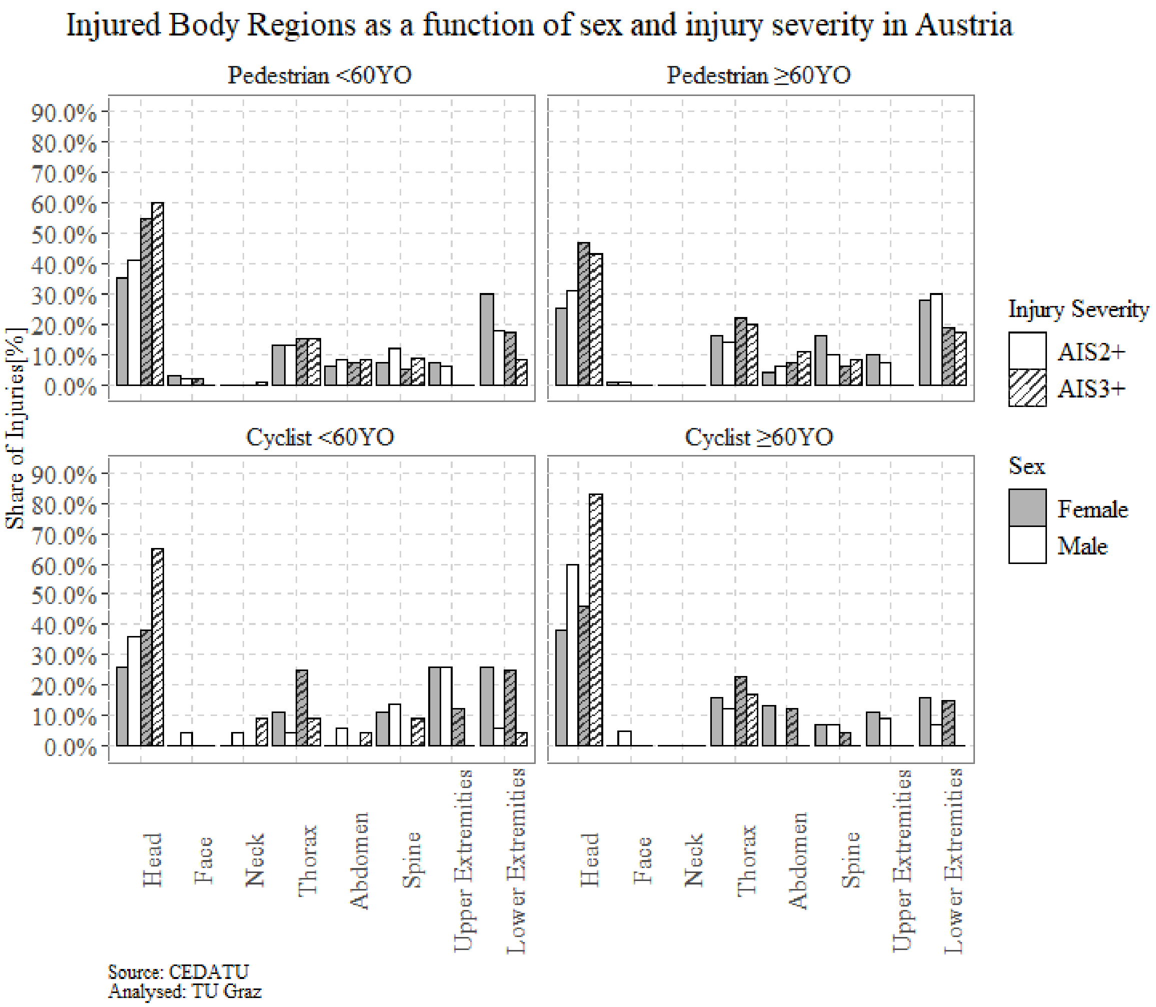

Figure 1 Injured Body Regions as a function of sex and injury severity in Austria

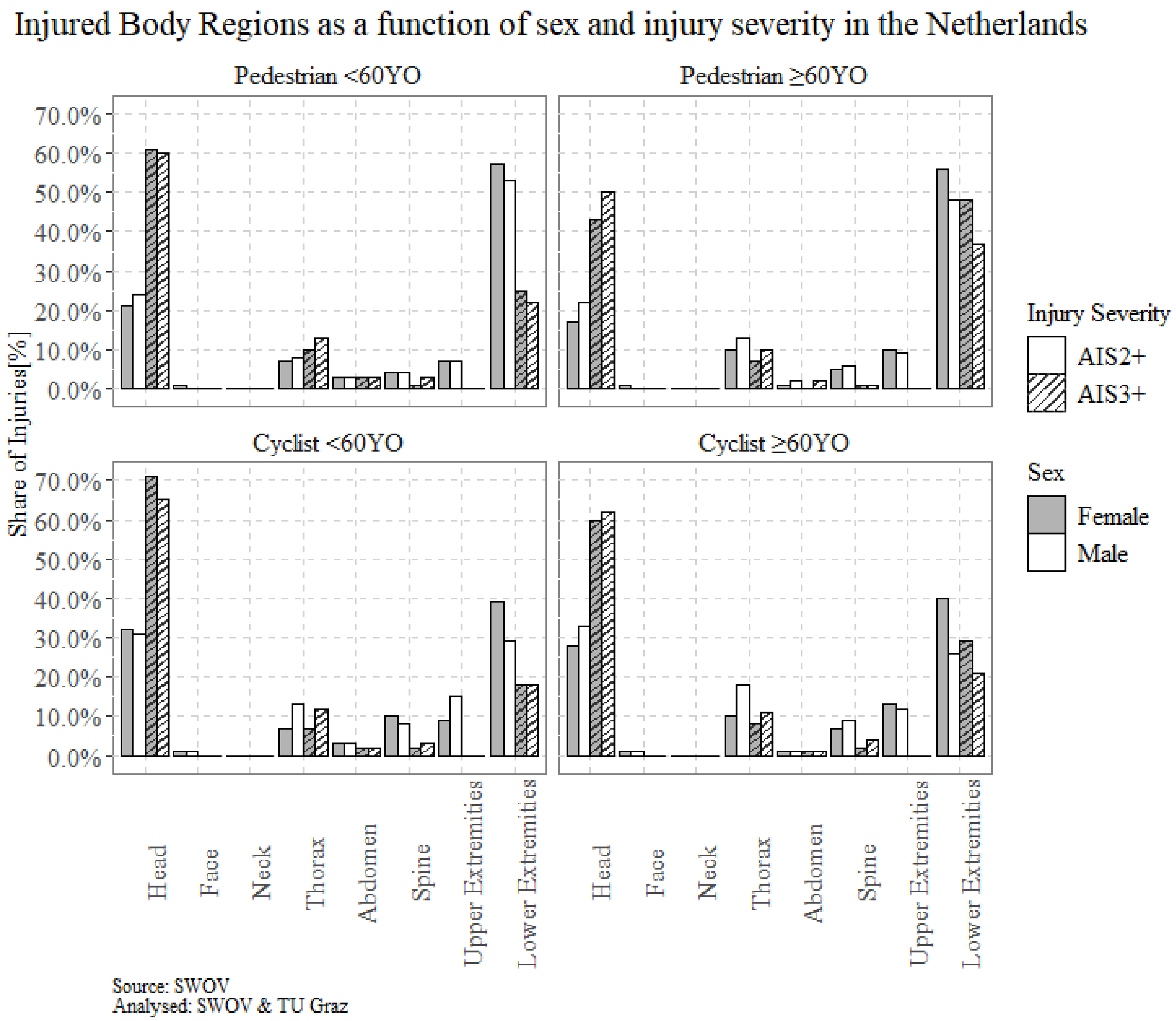

Figure 2 Injured Body Regions as a function of sex and injury severity in the Netherlands

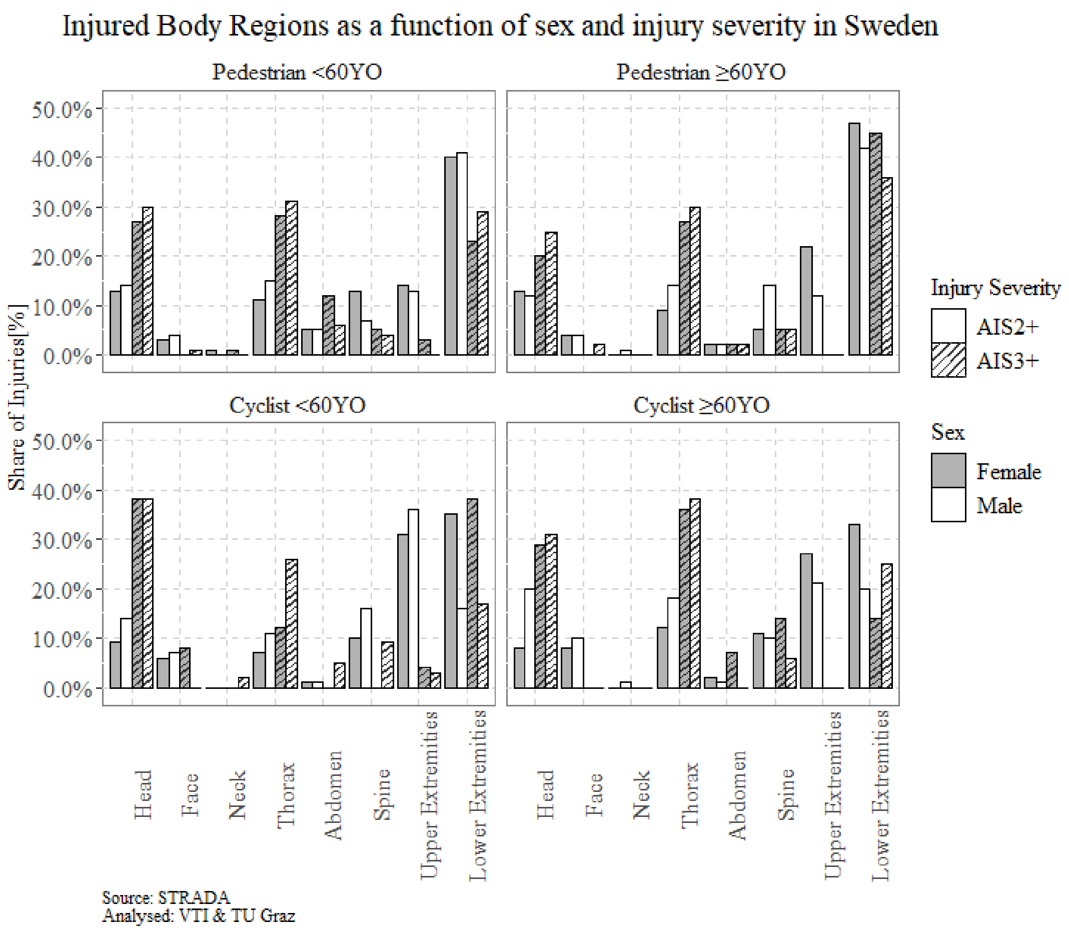

Figure 3 Injured Body Regions as a function of sex and injury severity in Sweden

Table 13 Injury Severity, OR and p-value for pedestrians and cyclists for different age groups in Austrian, Dutch and Swedish accident data (*p-value <5%)

Injury Severity in Austria

Severity	≥60YO	<60YO	OR	95%-CI	p-value
**Pedestrian**	*n=546*	*n=578*			
AIS1+	100.0%	100.0%	-	-	-
AIS2+	75.6%	73.0%	0.87	[0.67; 1.14]	0.313
AIS3+	34.4%	37.4%	1.14	[0.89; 1.45]	0.305
AIS4+	12.6%	15.2%	1.24	[0.88; 1.75]	0.211
**Cyclist**	*n=136*	*n=164*			
AIS1+	100.0%	100.0%	-	-	-
AIS2+	64.7%	42.1%	0.4	[0.25; 0.63]	<0.001*
AIS3+	36.0%	18.9%	0.42	[0.24; 0.7]	0.001*
AIS4+	12.5%	7.3%	0.56	[0.25; 1.21]	0.130

Visualization (Pedestrian):

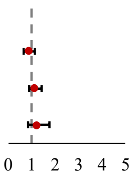

Visualization (Cyclist):

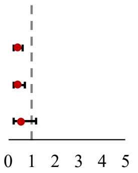

Injury Severity in the Netherlands

Severity	≥60YO	<60YO	OR	95%-CI	p-value
**Pedestrian**	*n=2584*	*n=5813*			
AIS1+	100.0%	100.0%	-	-	-
AIS2+	72.4%	65.3%	0.72	[0.65; 0.79]	<0.001*
AIS3+	22.9%	17.9%	0.73	[0.65; 0.82]	<0.001*
AIS4+	0.4%	0.9%	2.09	[1.13; 4.24]	0.022*
**Cyclist**	*n=8859*	*n=15703*			
AIS1+	100.0%	100.0%	-	-	-
AIS2+	66.6%	58.5%	0.71	[0.67; 0.75]	<0.001*
AIS3+	24.6%	18.7%	0.7	[0.66; 0.75]	<0.001*
AIS4+	0.7%	0.6%	0.81	[0.59; 1.13]	0.214

Visualization (Pedestrian):

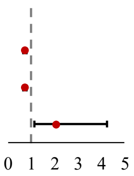

Visualization (Cyclist):

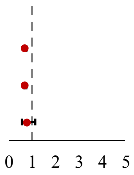

Injury Severity in Sweden

Severity	≥60YO	<60YO	OR	95%-CI	p-value
**Pedestrian**	*n=1033*	*n=2165*			
AIS1+	100.0%	100.0%	-	-	-
AIS2+	43.9%	31.4%	0.58	[0.5; 0.68]	<0.001*
AIS3+	9.6%	8.7%	0.9	[0.7; 1.16]	0.405
AIS4+	2.8%	3.3%	1.17	[0.76; 1.84]	0.473
**Cyclist**	*n=889*	*n=2951*			
AIS1+	100.0%	100.0%	-	-	-
AIS2+	35.8%	20.6%	0.47	[0.39; 0.55]	<0.001*
AIS3+	7.0%	3.1%	0.42	[0.3; 0.59]	<0.001*
AIS4+	0.7%	0.9%	1.33	[0.58; 3.62]	0.497

Visualization (Pedestrian):

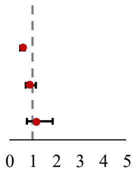

Visualization (Cyclist):

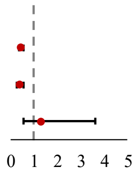

Table 14 Accident Location, OR and p-value for pedestrians and cyclists <60YO and ≥60YO in Austrian, Dutch and Swedish accident data (*p-value <5%)

Accident Location in Austria

Location	Male	Female	OR	95%-CI	p-value
**Pedestrian <60YO**	*n=147*	*n=44*			
Urban	56.5%	68.2%	1.6	[0.81; 3.45]	0.165
Rural	43.5%	31.8%	0.6	[0.29; 1.23]	0.165
Unknown	0.0%	0.0%	-	-	-
**Pedestrian ≥60YO**	*n=72*	*n=55*			
Urban	47.2%	85.5%	6.4	[2.74; 16.52]	<0.001*
Rural	52.8%	14.5%	0.2	[0.06; 0.36]	<0.001*
Unknown	0.0%	0.0%	-	-	-
**Cyclist <60YO**	*n=54*	*n=25*			
Urban	61.1%	80.0%	2.5	[0.84; 8.55]	0.097
Rural	38.9%	20.0%	0.4	[0.12; 1.19]	0.097
Unknown	0.0%	0.0%	-	-	-
**Cyclist ≥60YO**	*n=32*	*n=19*			
Urban	37.5%	47.4%	1.5	[0.46; 4.84]	0.489
Rural	62.5%	52.6%	0.7	[0.21; 2.18]	0.489
Unknown	0.0%	0.0%	-	-	-

Visualisation

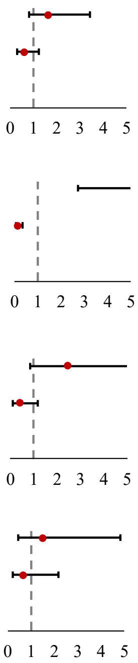

Accident Location in the Netherlands

Location	Male	Female	OR	95%-CI	p-value
**Pedestrian <60YO**	*n=1580*	*n=941*			
Urban	82.0%	85.4%	1.29	[1.03; 1.61]	0.026*
Rural	14.2%	10.6%	0.72	[0.56; 0.92]	0.009*
Unknown	3.7%	3.9%	1.06	[0.69; 1.6]	0.802
**Pedestrian ≥60YO**	*n=411*	*n=698*			
Urban	85.2%	89.0%	1.41	[0.98; 2.02]	0.063
Rural	8.3%	5.0%	0.59	[0.36; 0.96]	0.030*
Unknown	6.6%	6.0%	0.91	[0.55; 1.52]	0.713
**Cyclist <60YO**	*n=3755*	*n=3319*			
Urban	74.9%	81.0%	1.43	[1.28; 1.6]	<0.001*
Rural	19.1%	12.7%	0.62	[0.54; 0.7]	<0.001*
Unknown	6.0%	6.3%	1.05	[0.87; 1.28]	0.594
**Cyclist ≥60YO**	*n=1774*	*n=1879*			
Urban	72.7%	80.7%	1.57	[1.35; 1.84]	<0.001*
Rural	20.9%	11.9%	0.51	[0.43; 0.61]	<0.001*
Unknown	6.5%	7.5%	1.16	[0.9; 1.5]	0.251

Visualization

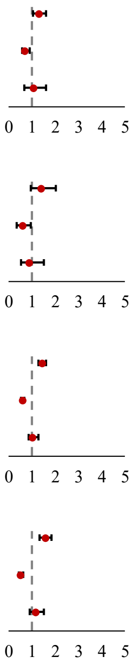

Accident Location in Sweden

Location	Male	Female	OR	95%-CI	p-value
**Pedestrian <60YO**	*n=415*	*n=509*			
Urban	77.6%	80.6%	1.2	[0.87; 1.65]	0.270
Rural	12.8%	8.1%	0.6	[0.39; 0.92]	0.018*
Unknown	9.6%	11.4%	1.2	[0.79; 1.86]	0.388
**Pedestrian ≥60YO**	*n=156*	*n=231*			
Urban	78.8%	88.7%	2.11	[1.2; 3.73]	0.008*
Rural	10.3%	4.8%	0.44	[0.19; 0.97]	0.037*
Unknown	10.9%	6.5%	0.57	[0.27; 1.19]	0.123
**Cyclist <60YO**	*n=762*	*n=782*			
Urban	80.3%	83.6%	1.25	[0.97; 1.63]	0.090
Rural	10.1%	7.2%	0.69	[0.48; 0.98]	0.039*
Unknown	9.6%	9.2%	0.96	[0.68; 1.35]	0.802
**Cyclist ≥60YO**	*n=218*	*n=170*			
Urban	77.1%	81.2%	1.28	[0.78; 2.13]	0.325
Rural	15.6%	8.8%	0.53	[0.27; 0.99]	0.046*
Unknown	7.3%	10.0%	1.4	[0.68; 2.9]	0.351

Visualisation

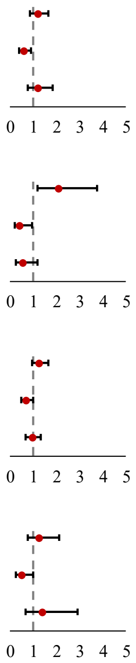

Table 15 Accident Severity, OR and p-value in Austria for pedestrians and cyclists <60YO and ≥60YO (*p-value <5%)

Accident Severity in Austria

Accident Severity	Male	Female	OR	95%-CI	p-value	Visualization
		**Pedestrian <60YO**				
	n=145	*n=44*				
slight	6.9%	13.6%	2.1	[0.68; 6.25]	0.16	
serious	27.6%	22.7%	0.8	[0.34; 1.69]	0.522	
fatal	65.5%	63.6%	0.9	[0.46; 1.89]	0.819	
		**Pedestrian ≥60YO**				
	n=72	*n=53*				
slight	4.2%	1.9%	0.5	[0.02; 4.29]	0.474	
serious	8.3%	24.5%	3.5	[1.26; 10.84]	0.013*	
fatal	87.5%	73.6%	0.4	[0.15; 1.02]	0.047	
		**Cyclist <60YO**				
	n=53	*n=23*				
slight	15.1%	30.4%	2.4	[0.73; 8.03]	0.123	
serious	34.0%	47.8%	1.8	[0.64; 4.89]	0.253	
fatal	50.9%	21.7%	0.3	[0.08; 0.82]	0.018*	
		**Cyclist ≥60YO**				
	n=32	*n=18*				
slight	3.1%	11.1%	3.6	[0.27; 117.57]	0.254	
serious	6.2%	22.2%	4	[0.66; 35.71]	0.095	
fatal	90.6%	66.7%	0.2	[0.04; 1]	0.034*	

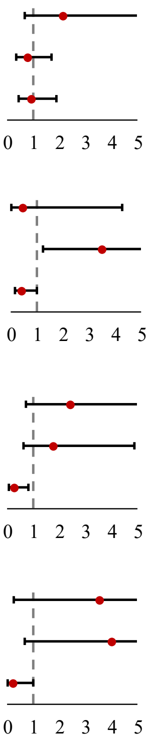

Table 16 MAIS, OR and p-value in Austria for pedestrians and cyclists <60YO and ≥60YO (*p-value <5%)

MAIS in Austria

MAIS	Male	Female	OR	95%-CI	p-value	Visualization
		**Pedestrian <60YO**				
	n=111	*n=39*				
MAIS1	17.1%	15.4%	0.89	[0.3; 2.35]	0.803	
MAIS2	21.6%	20.5%	0.95	[0.36; 2.27]	0.884	
MAIS3	37.8%	23.1%	0.5	[0.2; 1.13]	0.094	
MAIS4	10.8%	35.9%	4.55	[1.86; 11.34]	<0.001*	
MAIS5	3.6%	5.1%	1.49	[0.18; 8.46]	0.676	
MAIS6	9.0%	0.0%	-	-	-	
		**Pedestrian ≥60YO**				
	n=50	*n=46*				
MAIS1	10.0%	4.3%	0.43	[0.05; 2.21]	0.287	
MAIS2	8.0%	17.4%	2.36	[0.67; 9.76]	0.165	
MAIS3	38.0%	37.0%	0.96	[0.41; 2.21]	0.916	
MAIS4	18.0%	28.3%	1.78	[0.67; 4.86]	0.232	
MAIS5	16.0%	4.3%	0.25	[0.03; 1.12]	0.062	
MAIS6	10.0%	8.7%	0.86	[0.19; 3.61]	0.827	
		**Cyclist <60YO**				
	n=31	*n=20*				
MAIS1	25.8%	25.0%	0.97	[0.24; 3.56]	0.949	
MAIS2	54.8%	40.0%	0.56	[0.17; 1.75]	0.301	
MAIS3	3.2%	25.0%	8.69	[1.2; 244.82]	0.018*	
MAIS4	0.0%	10.0%	-	-	-	
MAIS5	6.5%	0.0%	-	-	-	
MAIS6	9.7%	0.0%	-	-	-	
		**Cyclist ≥60YO**				
	n=10	*n=14*				
MAIS1	20.0%	21.4%	1.07	[0.13; 10.97]	0.932	
MAIS2	10.0%	21.4%	2.21	[0.21; 71.34]	0.459	
MAIS3	50.0%	21.4%	0.29	[0.04; 1.75]	0.143	
MAIS4	20.0%	7.1%	0.34	[0.01; 4.84]	0.348	
MAIS5	0.0%	14.3%	-	-	-	
MAIS6	0.0%	14.3%	-	-	-	

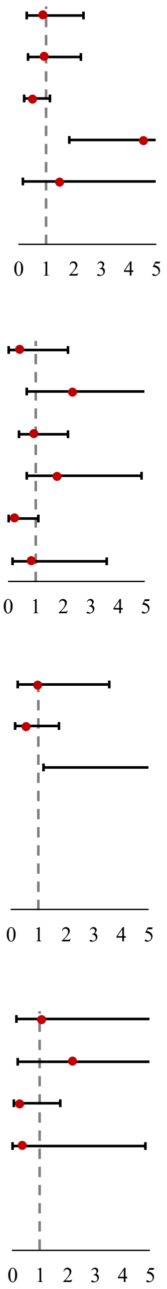

Table 17 Road conditions, OR and p-value for pedestrians and cyclists <60YO and ≥60YO in Austrian, and Dutch accident data (*p-value <5%)

Road Conditions in Austria

Road Conditions	Male	Female	OR	95%-CI	p-value	Visualization
	**Pedestrian <60YO**					
	n=147	*n=44*				
dry	75.5%	72.7%	0.86	[0.41; 1.91]	0.709	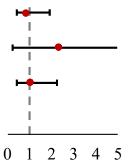
slippery	2.0%	4.5%	2.33	[0.26; 15.76]	0.361	
wet	22.4%	22.7%	1.02	[0.44; 2.24]	0.969	
unknown	0.0%	0.0%	-	-	-	
	**Pedestrian ≥60YO**					
	n=72	*n=53*				
dry	69.4%	66.0%	0.86	[0.4; 1.85]	0.687	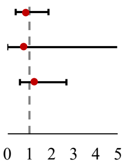
slippery	2.8%	1.9%	0.72	[0.02; 9.08]	0.748	
wet	27.8%	32.1%	1.23	[0.56; 2.68]	0.603	
unknown	0.0%	0.0%	-	-	-	
	**Cyclist <60YO**					
	n=52	*n=25*				
dry	94.2%	96.0%	1.35	[0.15; 40.21]	0.743	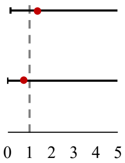
slippery	0.0%	0.0%	-	-	-	
wet	5.8%	4.0%	0.74	[0.02; 6.76]	0.743	
unknown	0.0%	0.0%	-	-	-	
	**Cyclist ≥60YO**					
	n=32	*n=19*				
dry	78.1%	94.7%	4.43	[0.68; 120.33]	0.115	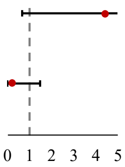
slippery	0.0%	0.0%	-	-	-	
wet	21.9%	5.3%	0.23	[0.01; 1.48]	0.115	
unknown	0.0%	0.0%	-	-	-	

Road Conditions in the Netherlands

Road Conditions	Male	Female	OR	95%-CI	p-value	Visualization
	**Pedestrian <60YO**					
	n=1580	*n=941*				
dry	72.4%	70.6%	0.91	[0.76; 1.09]	0.321	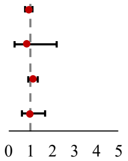
slippery	0.8%	0.6%	0.85	[0.29; 2.22]	0.725	
wet	24.2%	26.1%	1.11	[0.92; 1.34]	0.27	
unknown	2.7%	2.7%	1	[0.6; 1.65]	0.998	
	**Pedestrian ≥60YO**					
	n=411	*n=698*				
dry	62.8%	65.0%	1.1	[0.86; 1.42]	0.446	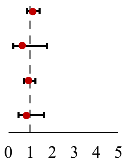
slippery	1.9%	1.3%	0.66	[0.25; 1.79]	0.39	
wet	31.1%	30.1%	0.95	[0.73; 1.24]	0.712	
unknown	4.1%	3.6%	0.86	[0.46; 1.64]	0.64	
	**Cyclist <60YO**					
	n=3755	*n=3319*				
dry	69.5%	68.4%	0.95	[0.86; 1.05]	0.338	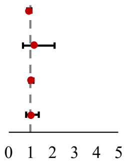
slippery	0.6%	0.8%	1.18	[0.67; 2.08]	0.564	
wet	27.1%	28.0%	1.04	[0.94; 1.16]	0.439	
unknown	2.7%	2.9%	1.04	[0.79; 1.39]	0.761	
	**Cyclist ≥60YO**					
	n=1774	*n=1879*				
dry	76.3%	77.7%	1.08	[0.93; 1.27]	0.304	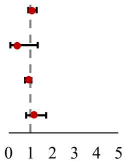
slippery	0.5%	0.2%	0.43	[0.11; 1.34]	0.135	
wet	20.6%	19.0%	0.91	[0.77; 1.07]	0.232	
unknown	2.6%	3.1%	1.17	[0.79; 1.74]	0.429	

Table 18 Light Conditions, OR and p-value for pedestrian and cyclists <60YO and ≥60YO in Austrian, and Dutch accident data (*p-value <5%)

Light Conditions in Austria

Light Conditions	Male	Female	OR	95%-CI	p-value	Visualization
		**Pedestrian <60YO**				
	n=146	*n=44*				
darkness	42.5%	25.0%	0.46	[0.2; 0.95]	0.037*	
dawn/twilight	3.4%	11.4%	3.58	[0.92; 13.96]	0.039*	
daylight	33.6%	40.9%	1.37	[0.68; 2.74]	0.371	
electric light	17.8%	20.5%	1.2	[0.49; 2.73]	0.691	
sudden change	2.7%	2.3%	0.91	[0.03; 6.78]	0.865	
unknown	0.0%	0.0%	-	-	-	
		**Pedestrian ≥60YO**				
	n=72	*n=55*				
darkness	43.1%	20.0%	0.34	[0.14; 0.74]	0.006*	
dawn/twilight	8.3%	1.8%	0.23	[0.01; 1.45]	0.111	
daylight	27.8%	41.8%	1.86	[0.88; 3.96]	0.098	
electric light	19.4%	30.9%	1.84	[0.81; 4.25]	0.136	
sudden change	1.4%	5.5%	3.73	[0.42; 109.21]	0.194	
unknown	0.0%	0.0%	-	-	-	
		**Cyclist <60YO**				
	n=54	*n=24*				
darkness	7.4%	0.0%	-	-	-	
dawn/twilight	0.0%	4.2%	-	-	-	
daylight	85.2%	83.3%	0.86	[0.23; 3.66]	0.834	
electric light	3.7%	4.2%	1.2	[0.04; 15.46]	0.922	
sudden change	3.7%	8.3%	2.33	[0.23; 23.62]	0.392	
unknown	0.0%	0.0%	-	-	-	
		**Cyclist ≥60YO**				
	n=32	*n=19*				
darkness	3.1%	10.5%	3.35	[0.25; 110.67]	0.277	
dawn/twilight	3.1%	0.0%	-	-	-	
daylight	65.6%	73.7%	1.44	[0.41; 5.57]	0.549	
electric light	18.8%	10.5%	0.54	[0.07; 2.76]	0.435	
sudden change	9.4%	5.3%	0.59	[0.02; 5.5]	0.597	
unknown	0.0%	0.0%	-	-	-	

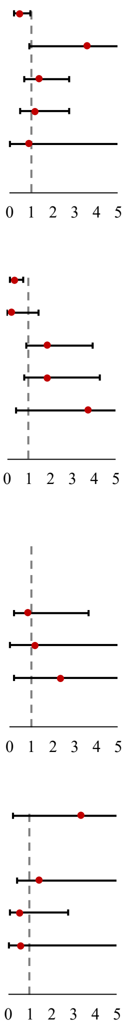

Light Conditions in the Netherlands

Light Conditions	Male	Female	OR	95%-CI	p-value	Visualization
		**Pedestrian <60YO**				
	n=1580	*n=941*				
darkness	28.5%	21.7%	0.7	[0.57; 0.84]	<0.001*	
dawn/twilight	5.0%	7.1%	1.46	[1.04; 2.04]	0.028*	
daylight	58.1%	59.7%	1.07	[0.91; 1.26]	0.424	
electric light	0.0%	0.0%	-	-	-	
sudden change	0.0%	0.0%	-	-	-	
unknown	8.4%	11.5%	1.41	[1.08; 1.84]	0.012*	
		**Pedestrian ≥60YO**				
	n=411	*n=698*				
darkness	27.5%	17.3%	0.55	[0.41; 0.74]	<0.001*	
dawn/twilight	4.4%	6.0%	1.39	[0.8; 2.51]	0.244	
daylight	52.6%	59.7%	1.34	[1.05; 1.71]	0.020*	
electric light	0.0%	0.0%	-	-	-	
sudden change	0.0%	0.0%	-	-	-	
unknown	15.6%	16.9%	1.1	[0.79; 1.54]	0.562	
		**Cyclist <60YO**				
	n=3755	*n=3319*				
darkness	20.9%	13.5%	0.59	[0.52; 0.67]	<0.001*	
dawn/twilight	6.3%	6.3%	1	[0.83; 1.21]	0.983	
daylight	60.5%	66.9%	1.32	[1.2; 1.45]	<0.001*	
electric light	0.0%	0.0%	-	-	-	
sudden change	0.0%	0.0%	-	-	-	
unknown	12.3%	13.3%	1.1	[0.96; 1.27]	0.179	
		**Cyclist ≥60YO**				
	n=1774	*n=1879*				
darkness	6.4%	3.4%	0.51	[0.37; 0.7]	<0.001*	
dawn/twilight	2.5%	2.6%	1.03	[0.68; 1.56]	0.892	
daylight	72.9%	76.7%	1.22	[1.05; 1.42]	0.009*	
electric light	0.0%	0.0%	-	-	-	
sudden change	0.0%	0.0%	-	-	-	
unknown	18.1%	17.3%	0.95	[0.8; 1.12]	0.527	

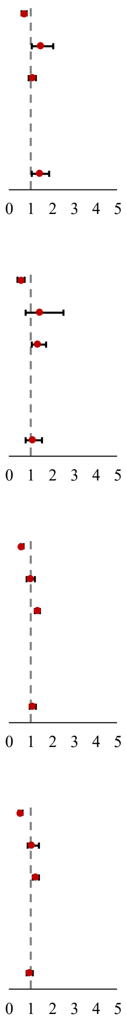

Table 19 Influence of alcohol, OR and p-value for pedestrian and cyclists <60YO and ≥60YO in Austrian accident data (*p-value<5%)

Alcohol Influence in Austria

Alcohol Influence	Male	Female	OR	95%-CI	p-value	Visualization
	**Pedestrian <60YO**					
	n=95	*n=28*				
yes	58.9%	21.4%	0.2	[0.07; 0.5]	<0.001*	
no	41.1%	78.6%	5.1	[1.98; 15.19]	<0.001*	
	**Pedestrian ≥60YO**					
	n=35	*n=18*				
yes	60.0%	27.8%	0.3	[0.07; 0.89]	0.026*	
no	40.0%	72.2%	3.7	[1.12; 14.29]	0.026*	
	**Cyclist <60YO**					
	n=26	*n=12*				
yes	0.0%	0.0%	-	-	-	
no	100.0%	100.0%	-	-	-	
	**Cyclist ≥60YO**					
	n=5	*n=3*				
yes	0.0%	0.0%	-	-	-	
no	100.0%	100.0%	-	-	-	

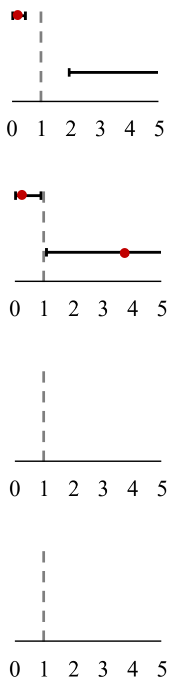

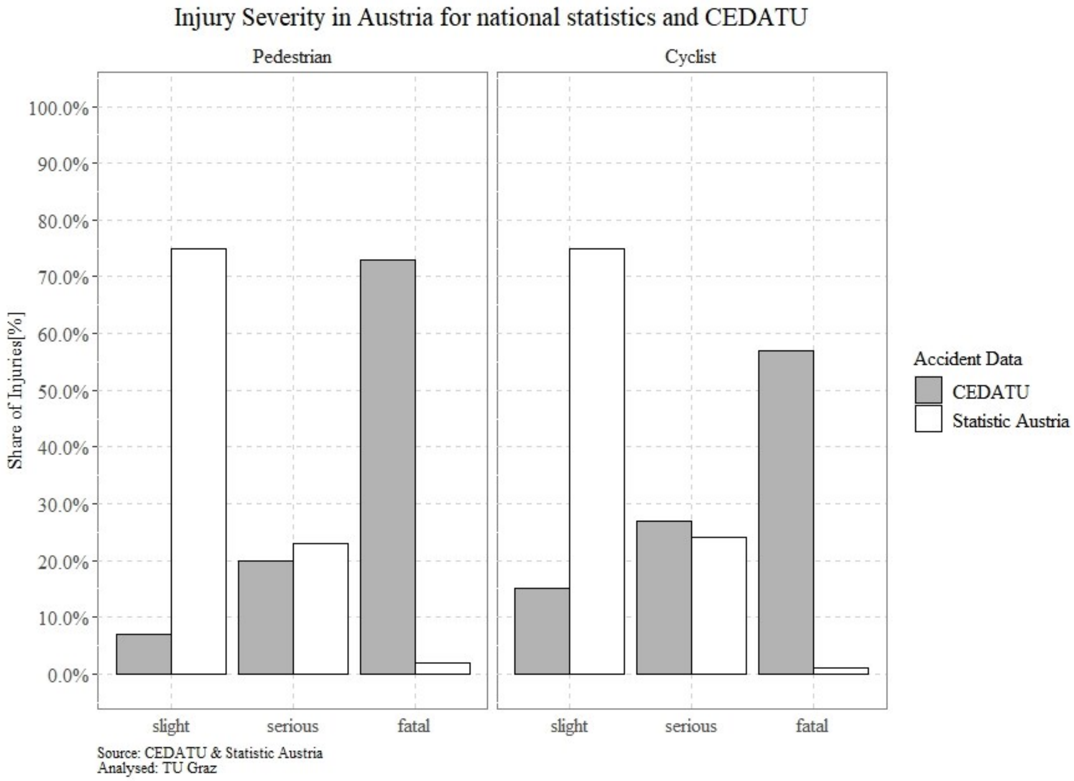

Figure 6 Share of injuries according to injury severity in Austria for national statistics and CEDATU
