# Supplementary figures and images for: Are There Any Significant Differences in Terms of Age and Sex in Pedestrian and Cyclist Accidents?
Source: Front Bioeng Biotechnol. 2021 May 24;9:677952. doi: 10.3389/fbioe.2021.677952 (PMC8183819; doi:10.3389/fbioe.2021.677952)

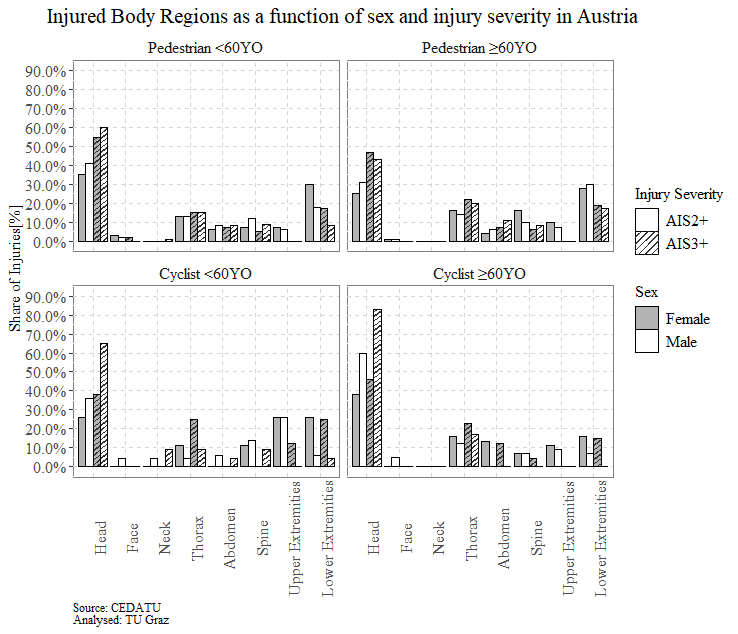

Supplement: Supplementary file 2 [file Image_1.TIFF]

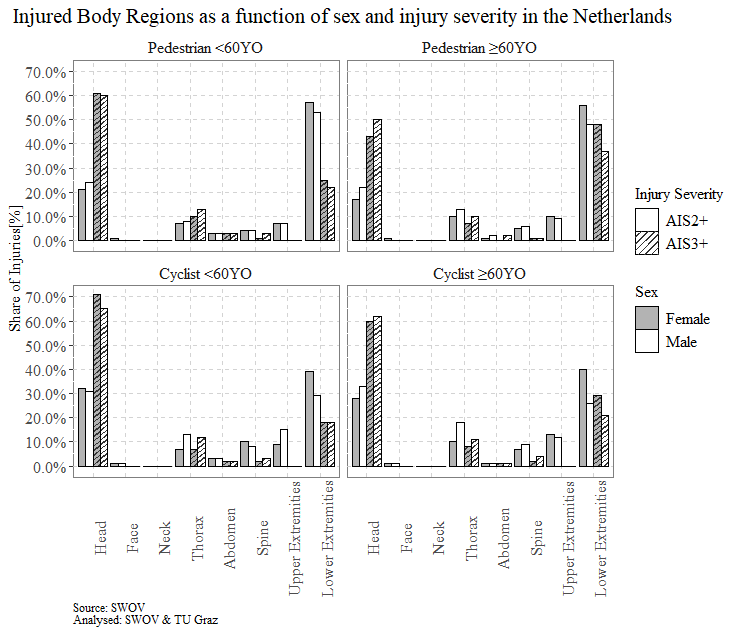

Supplement: Supplementary file 3 [file Image_2.TIFF]

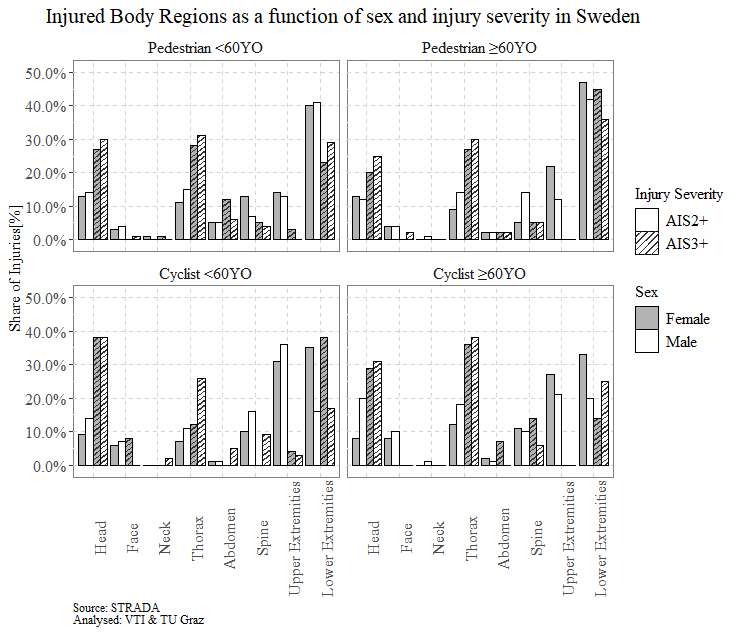

Supplement: Supplementary file 4 [file Image_3.TIFF]

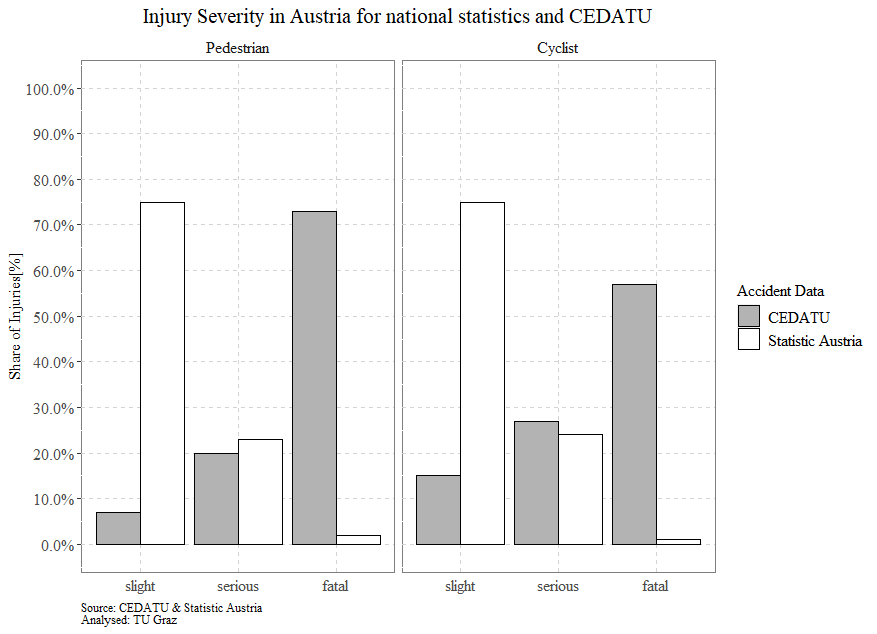

Supplement: Supplementary file 5 [file Image_4.TIFF]
